# Isocitrate dehydrogenase 2 protects mice from high-fat diet-induced metabolic stress by limiting oxidative damage to the mitochondria from brown adipose tissue

**DOI:** 10.1038/s12276-020-0379-z

**Published:** 2020-02-03

**Authors:** Jae-Ho Lee, Younghoon Go, Do-Young Kim, Sun Hee Lee, Ok-Hee Kim, Yong Hyun Jeon, Taeg Kyu Kwon, Jae-Hoon Bae, Dae-Kyu Song, Im Joo Rhyu, In-Kyu Lee, Minho Shong, Byung-Chul Oh, Christopher Petucci, Jeen-Woo Park, Timothy F. Osborne, Seung-Soon Im

**Affiliations:** 1 Department of Physiology, Keimyung University School of Medicine, Daegu, 42601 Republic of Korea; 2 Department of Internal Medicine, School of Medicine Kyungpook National University, Kyungpook National University Hospital, Daegu, 41944 Republic of Korea; 3 Leading-Edge Research Center for Drug Discovery and Development for Diabetes and Metabolic Disease, Kyungpook National University Hospital, Daegu, 41404 South Korea; 4 Korean Medicine Application Center, Korea Institute of Oriental Medicine, Daegu, 41062 Republic of Korea; 5 Department of Physiology, Lee Gil Ya Cancer and Diabetes Institute, Gachon University School of Medicine, Younsoo-gu, Inchon, 21999 Republic of Korea; 6 Laboratory Animal Center, Daegu-Gyeongbuk Medical Innovation Foundation, Daegu, 41061 Republic of Korea; 7 Department of Immunology, Keimyung University School of Medicine, Daegu, 42601 Republic of Korea; 8 Department of Anatomy, Korea University College of Medicine, Seoul, 02841 Republic of Korea; 9 Research Center for Endocrinology and Metabolism, Chungnam National University Hospital (CNUH), 282 Munhwaro, Daejeon, 35015 Republic of Korea; 10 Center for Metabolic Origins of Disease, Sanford Burnham Prebys Medical Discovery Institute, Orlando, FL 32827 USA; 11 Cardiovascular Institute and Department of Medicine, Perelman School of Medicine at the University of Pennsylvania, Philadelphia, PA 19104 USA; 12 School of Life Sciences and Biotechnology, College of Natural Science, Kyungpook National University, Daegu, 41566 Republic of Korea; 13 Institute for Fundamental Biomedical Research, Department of Medicine and Biological Chemistry, Johns Hopkins University School of Medicine, St. Petersburg, FL 33701 USA

**Keywords:** Obesity, Metabolic syndrome

## Abstract

Isocitrate dehydrogenase 2 (IDH2) is an NADP^+^-dependent enzyme that catalyzes the oxidative decarboxylation of isocitrate to α-ketoglutarate in the mitochondrial matrix, and is critical for the production of NADPH to limit the accumulation of mitochondrial reactive oxygen species (ROS). Here, we showed that high-fat diet (HFD) feeding resulted in accelerated weight gain in the IDH2KO mice due to a reduction in whole-body energy expenditure. Moreover, the levels of NADP^+^, NADPH, NAD^+^, and NADH were significantly decreased in the brown adipose tissue (BAT) of the HFD-fed IDH2KO animals, accompanied by decreased mitochondrial function and reduced expression of key genes involved in mitochondrial biogenesis, energy expenditure, and ROS resolution. Interestingly, these changes were partially reversed when the antioxidant butylated hydroxyanisole was added to the HFD. These observations reveal a crucial role for IDH2 in limiting ROS-dependent mitochondrial damage when BAT metabolism is normally enhanced to limit weight gain in response to dietary caloric overload.

## Obesity: Possible protective enzyme identified

An enzyme that limits the build-up of reactive oxygen species (ROS) in fat cells protects mice against metabolic stress during a high-fat diet. Calorie overload leads to high levels of damaging ROS in the mitochondria of brown fat cells. This can disrupt processes that regulate energy expenditure and glucose metabolism. A team led by Seung-Soon Im at Keimyung University, Daegu, South Korea, and Timothy F. Osborne at Johns Hopkins University, St. Petersburg, USA, examined the role of an enzyme called isocitrate dehydrogenase 2 (IDH2), which is known to regulate the build-up of mitochondrial ROS. In mice fed a high-fat diet, those without IDH2 experienced accelerated weight gain, triggered by increased ROS levels and decreased mitochondrial function. A dose of an antioxidant in the diet reduced this effect, suggesting that patients with obesity may benefit from antioxidant supplements.

## Introduction

Brown adipose tissue (BAT) is a major metabolic organ in mammals, and has a key role in regulating whole-body thermogenesis, energy expenditure, and glucose metabolism^1^. Uncoupled respiration in the BAT is increased to prevent weight gain and insulin resistance when mice are fed a high-fat diet (HFD)^2^. A potentially unhealthy complication of elevated uncoupled respiration is the generation of mitochondrial reactive oxygen species (ROS), which must be limited to prevent oxidative damage to the enzyme complexes of the electron transport (ET) chain and the tricarboxylic acid (TCA) cycle^3,4^.

Proper coordinated action of key mitochondrial ET and TCA enzymes is required to modulate substrate flow and balance electron transfer. One of the key steps in the TCA cycle is the oxidative decarboxylation of isocitrate to generate α-ketoglutarate (αKG), which is catalyzed by isocitrate dehydrogenase (IDH) in the mitochondrial matrix. There are three mammalian IDH isoforms (IDH1, IDH2, and IDH3). The reaction catalyzed by IDH3 is irreversible, and this protein is thought to be the isoform responsible for the bulk of carbon flux through the TCA cycle, in which the electron capture is paired with the nicotinamide adenine dinucleotide (NAD^+^) and nicotinamide adenine dinucleotide hydrogen (NADH) cofactors. In contrast, both IDH1 and IDH2 catalyze reversible reactions, are paired with nicotinamide adenine dinucleotide phosphate (NADP^+^) and nicotinamide adenine dinucleotide hydrogen phosphate (NADPH), and reside in the cytosol and mitochondrial matrix, respectively. IDH1 and IDH2 have been proposed to balance cytoplasmic and mitochondrial redox, respectively, as their major roles^5^. Mitochondrial NADPH is essential to provide reducing equivalents to maintain glutathione (GSH) for the glutathione peroxidase (GPX)-dependent scavenging of mitochondrial ROS, which protects against the oxidative damage that occurs during times of elevated ET chain activity^6,7^.

Although IDH2 is considered a unique enzyme in the regulation of mitochondrial ROS, its contribution to mitochondrial function during metabolic stresses such as HFD-dependent obesity is unknown. In this study, we showed that HFD-challenged IDH2KO mice gained significantly more weight than WT mice fed the same diet. The excess weight gains in the IDH2KO group occurred more rapidly than those in the WT group, and were accompanied by increased levels of cellular ROS and reduced energy expenditure in the BAT. The excess weight gain and impaired BAT activity were reversed when the antioxidant butylated hydroxyanisole (BHA) was added to the HFD. These studies uncover a critical role for IDH2 in balancing the ROS levels in the BAT when defending against body weight gain in response to the metabolic challenge of excess calorie consumption.

## Materials and methods

### Animal studies

All procedures were performed in accordance with the Institutional Animal Care and Use Committees at Keimyung University School of Medicine, Daegu, South Korea (KM-2015-32R3). Four-week-old male IDH2KO mice^8^ and WT littermates with the same genetic background (C57BL/6J) were used for this study. No randomization of the mice was used. All animals were kept under 12-h light–dark cycles (6 a.m.–6 p.m. light, 6 p.m.–6 a.m. light) at 22–24 °C and 60–70% humidity with free access to water in a specific pathogen-free facility. These mice were fed either a low-fat diet (LFD, Research Diet: D12450J, containing 10% fat [kcal%]) or a HFD (Research Diet: D12492, containing 60% fat [kcal%]), and 7.5 g/kg BHA (Sigma Aldrich, St. Louis, Missouri, USA) using a modified protocol from a previous study^9^. We mixed the purchased HFD and LFD with butylated hydroxyanisole by grinding the BHA together with the HFD or LFD pellets, and provided the combined mixture to the different groups of mice. Body weight was monitored weekly. Body composition was determined by nuclear magnetic resonance (NMR, LF50 BCA-Analyzer, Bruker, Brussels, Belgium). After 4 weeks on the specific diets, the mice were killed by cervical dislocation at 9:00 a.m.

### Preparation of fatty acid solutions

Solutions of 150 mM sodium palmitate were prepared in 50% ethanol by heating at 65 °C and vortexing until dissolved. These solutions were diluted 20-fold in a 10% fatty acid-free, low-endotoxin BSA solution to achieve a final molar ratio of 19:1. Conjugation was performed at 37 °C for 1 h. The BSA–fatty acid conjugates were further diluted 15-fold in cell culture media to reach a final concentration of 0.5 mM palmitate. Control BSA was prepared by adding the same amount of 50% ethanol to a 10% BSA solution. All preparations were aliquoted and frozen at −20 °C.

### Metabolic analysis

Mice were fed LFD and HFD when transferred to a single housing unit in the Phenomaster System (TSE Systems Gmbh, Bad Homburg, Germany) for 5 days (*n* = 6 per group). During the study period, airflow, temperature, oxygen and carbon dioxide content, oxygen uptake (VO_2_), and carbon dioxide production (VCO_2_) were measured simultaneously by using standard indirect calorimetry analysis. Energy expenditure and respiratory exchange ratio were calculated automatically from the VO_2_ and VCO_2_. The data were collected in TSE Phenomaster software and exported to Excel.

### Positron emission tomography/computed tomography imaging

Mice housed at room temperature (22–24 °C) and fed HFD for 4 weeks were used. For positron emission tomography/computed tomography (PET/CT) imaging, 20-min scans (DC imaging) were performed with the Triumph II PET-CT system (LabPET8; Gamma Medica-Ideas, Waukesha, WI, USA). For ^18^F-fluorodeoxyglucose (^18^F-FDG) PET/CT imaging, 10-min scans were performed with the same animal PET/CT as described above. CT scans were performed with an X-ray detector (fly acquisition; number of projections: 512; binning setting: 2 × 2; frame number: 1; X-ray tube voltage: 75 kVp; focal spot size: 50 μm; magnification factor: 1.5; matrix size: 512) immediately following the acquisition of PET images. PET images were reconstructed by three-dimensional (3D)-ordered subset expectation maximization iterative image reconstruction, and CT images were reconstructed using filtered back projections. All mice (*n* = 5 per genotype) were anesthetized using 1–2% isoflurane gas during imaging. PET images were coregistered with anatomical CT images using 3D image visualization and analysis software (VIVID; Gamma Medica-Ideas, Northridge, CA, USA). For determination of the uptake (%ID/cc) for the volumes of interest (VOIs), the VOIs from each image were manually segmented from the coregistered CT images using both VIVID and PMOD software (PMOD Technologies, Zurich, Switzerland), and the uptake in the region of interest was measured with PMOD 3.5 software.

### Isolation of primary brown adipocytes from the BAT

The primary brown adipocytes of the BAT from 4-week-old mice were separated by collagenase digestion. Briefly, the adipose tissues were dissected, minced, and digested with 0.2% collagenase type I (Worthington Biochemical Corp., Freehold, NJ, USA) in PBS buffer for 30 min at 37 °C. Mature adipocytes and connective tissues were separated from the cell pellet by centrifugation at 1000 × *g* for 5 min at 4 °C. The cell pellet was then suspended in FBS and filtered through a 40-μm mesh filter (BD Bioscience, San Jose, CA, USA). The pelleted primary brown adipocytes were resuspended in Dulbecco’s modified Eagle’s medium (DMEM) containing 15% FBS and seeded in six-well plates for proliferation. The cells were differentiated using a standard cocktail (DMEM containing 15% FBS, 50 nM insulin, 5 nM T3, and 1 μM rosiglitazone) for 2 days.

### Mitochondrial ROS production assay

Primary brown adipocytes (5 × 10^5^ cells/well) were cultured in the absence or presence of 500 μM palmitate. Then, 100 μM BHA was added and incubated for 1 h. In all, 5 μM MitoSOX^TM^ Red mitochondrial superoxide indicator (MitoSOX Red, Molecular Probes, Eugene, OR, USA) was added to the cells and incubated at 37 °C for 15 min. The cells were harvested by treatment with 0.05% trypsin, and then washed twice with cold PBS. The cells conjugated with MitoSOX Red (excitation, 510 nm; emission, 580 nm) were detected using the FL1 setting of a FACSCalibur system (BD Biosciences).

### Mitochondrial oxygen consumption rate

The mitochondrial oxygen consumption rate (OCR) was measured using a Seahorse XF-24 analyzer (Seahorse Bioscience, Inc., North Billerica, MA, USA) in 24-well plates. Primary brown adipocytes were seeded at 2 × 10^4^ cells per well and treated with 100 μM BHA before analysis. On the day before the OCR measurement, the sensor cartridge was placed into calibration buffer (Seahorse Bioscience) and incubated in a non-CO_2_ incubator at 37 °C. The primary brown adipocytes were washed and incubated in DMEM without sodium bicarbonate. The medium and mitochondrial OXPHOS inhibitors were adjusted to pH 7.4 on the day of the OCR assay. The basal OCR was measured three times, and three readings were taken after the addition of each mitochondrial OXPHOS inhibitor [oligomycin (2 µg/mL) and rotenone (1 µM)]. The basal and post-oligomycin OCRs were calculated by averaging the last three measurements after maintaining a steady state. Coupled respiration is expressed as the percent decrease from basal respiration. In addition, carbonyl cyanide m-chlorophenyl hydrazone (CCCP, 5 µM) was used to measure the maximal mitochondrial respiration of the cells. The OCR was automatically calculated and recorded by the sensor cartridge and Seahorse XF-24 software. After the assays, the plates were saved, and the protein levels were measured in each well to confirm equal cell numbers per well. The percentage of change compared with basal rates was calculated as the value of the change divided by the average value of baseline readings.

### Transmission electron microscopy

After collection, the mouse interscapuler BAT (iBAT) (*n* = 6 per genotype) was prefixed in 2% paraformaldehyde and 2.5% glutaraldehyde (Electron Microscopy Sciences, Fort Washington, PA, USA) in 0.1 M phosphate buffer (pH 7.4). The tissues were removed and stored in the same fresh fixative overnight at 4 °C. The tissues were washed, postfixed in 1% osmium tetroxide for 2 h, dehydrated through an ascending series of ethanol and propylene oxide, and embedded in Epon mixture (Oken Shoji Co., Tokyo, Japan). Thin sections (70 nm) were made using a Leica EM UC6 ultramicrotome (Leica Microsystems, Wetzlar, Germany), mounted on 200-mesh copper grids, stained with 2% uranyl acetate and 1% lead citrate for 5 min each, and observed under a Hitachi H-7650 transmission electron microscope (Hitachi, Tokyo, Japan) at an accelerating voltage of 80 kV.

### RNA sequencing

The BAT was harvested from the WT and IDH2KO mice fed an LFD or HFD (*n* = 9 per group). Equal amounts of RNA from three individual mice were pooled together to generate three separate samples for analysis (Macrogen, Inc., Seoul, Korea). Briefly, 1 μg of the total RNA was analyzed using the TruSeq RNA library kit to construct the cDNA libraries. The protocol included polyA-selected RNA extraction, RNA fragmentation, random hexamer-primed reverse transcription, and 100-nucleotide paired-end sequencing using an Illumina HiSeq 2000 (Illumina, San Diego, CA, USA). The libraries were quantified using quantitative real-time PCR (qPCR) according to the qPCR Quantification Protocol Guide. Agilent Technologies 2100 Bioanalyzer was used for the qualification.

### Immunoblotting

Whole-tissue lysate was extracted using RIPA lysis buffer (50 mM Tris-HCl, pH 8.0, 150 mM NaCl, 1.0% NP-40 or Triton X-100, 0.5% Na_3_VO_4_, and 0.1% SDS) supplemented with a complete protease inhibitor cocktail (GeneDepot, Barker, TX, USA). Proteins were diluted in 5× loading dye and heated at 94 °C for 5 min. The proteins were resolved by 5–10% Tris-HCl SDS/PAGE gel electrophoresis and transferred onto nitrocellulose membranes (GE Healthcare, Uppsala, Sweden). All immunoblots were developed by HRP-conjugated secondary antibody with an ECL detection system (AbFrontier, Seoul, Korea). Anti-PGC-1α (ab54481) and anti-catalase (ab16731) were purchased from Abcam (Cambridge, UK). SIRT1 (2028), SIRT2 (12650), SIRT3 (5490), acetylated lysine (9441), and SOD2 (13141) antibodies were purchased from Cell Signaling (Beverly, MA, USA). GPX3 (AG-25A-0073) antibody was purchased from Adipogen (San Diego, CA, USA). Anti-NAMPT (sc-393444) and anti-GAPDH (sc-47724) as the loading controls were purchased from Santa Cruz (Dallas, TX, USA).

### Measurement of mitochondrial DNA copy number

Total DNA was extracted with a DNA Isolation Kit (Bioseum, Seoul, South Korea). For measurement of the mitochondrial DNA (mtDNA) copy number, real-time PCR was performed using a CFX96 Bio-Rad qPCR machine (Bio-Rad, Richmond, CA, USA) utilizing cyclooxygenase 1 (COX1) primers for mtDNA, and normalized against the nuclear H-19 gene. The relative mtDNA copy number was defined as the total amount of mtDNA divided by the total amount of nuclear DNA.

### Sample preparation of tissue homogenates for targeted metabolomics and LC/MS/MS quantitation

Frozen brown adipose tissues from the WT and IDH2KO mice (*n* = 6 per group) were homogenized in ice-cold 50% acetonitrile/50% water with 0.3% formic acid. The tissue homogenates were aliquoted for the metabolomics assays and stored at −80 °C. For the analysis of organic acids, the homogenates (50 µL) were spiked with isotopically labeled internal standards and extracted in ethyl acetate, and organic acids in dried extracts were derivatized with O-benzylhydroxylamine using 1-ethyl-3-(3-dimethylaminopropyl)carbodiimide (EDC) coupling chemistry according to prior studies^10^. Then, the derivatized organic acids were quantitated using multiple reaction monitoring on a Dionex UltiMate 3000 HPLC/Thermo Scientific Quantiva triple- quadrupole mass spectrometer as previously described^10^.

For NADH and NADPH extraction, 100-µL aliquots of the homogenates were spiked with isotopically labeled internal standards. This procedure was followed by the addition of 100 µL of 1 M ammonium formate to adjust the pH to 4. The samples were vortexed thoroughly and centrifuged at 18,000 × *g* for 5 min at 10 °C. The clarified homogenates were passed through an AcroPrep Advance 3K Omega Filter Plate (Pall Corporation) prior to LC/MS/MS analysis. Quantitation of the reduced nucleotides was achieved using multiple reaction monitoring on a Dionex UltiMate 3000 HPLC/Thermo Scientific Quantiva triple- quadrupole mass spectrometer^10^.

### Statistical analysis

Densitometry was carried out using Image Studio version 5.2 (LI-COR). Data were analyzed using GraphPad Prism 7.0 for Windows (GraphPad Software). Student’s *t* test was two-tailed, and data are shown as the mean ± SEM. A *p* value <0.05 was considered significant; *p < 0.05, **p < 0.01, and ***p < 0.0005.

## Results

### IDH2KO mice are more susceptible to HFD-induced obesity than WT mice

**Fig. 1:**
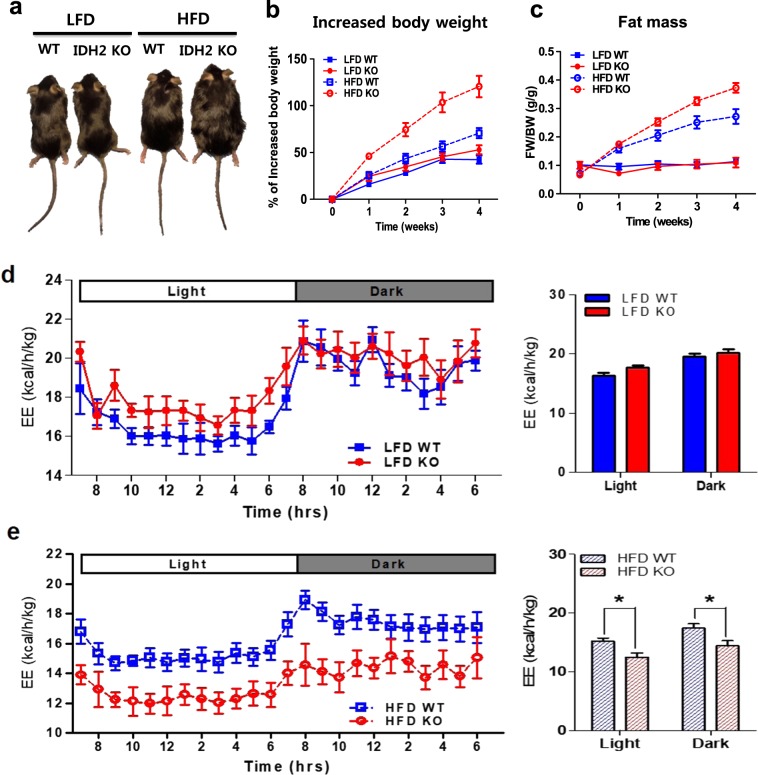
Impaired energy expenditure by a HFD in the IDH2KO mice compared with the WT mice. **a** Photographs of representative WT or IDH2KO mice after 4 weeks of HFD feeding. **b** Body weights in each group over a period of 4 weeks (*n* = 6 per group). **c** Time course of changes in fat mass. **d**, **e** Energy expenditure over a 24-h period of the WT and IDH2KO mice fed a LFD and HFD. *p < 0.05 vs. the HFD–WT mice. LFD low-fat diet, HFD high-fat diet, WT wild type, KO knockout, EE energy expenditure.

To explore the potential role of IDH2 in regulating whole-body energy homeostasis during metabolic stress, we compared the weight gain in the WT mice vs. the mice with global IDH2KO fed either a HFD or a matched LFD (Fig. 1). The IDH2KO mice fed the HFD rapidly gained weight, and by 4 weeks, they had almost doubled their body weight (Fig. 1a). In contrast, the HFD-fed WT mice gained significantly less weight and did so much more slowly than the IDH2KO mice (Fig. 1b). There was no difference between the LFD-fed mice of either genotype. Most of the excess weight in the HFD-fed IDH2KO mice was due to an increase in fat mass and a decrease in lean mass (Fig. 1 and Fig. S1a–d). The serum lipid levels were similarly elevated in the HFD-fed WT and IDH2KO mice; however, there was a significant increase in the serum alanine aminotransferase (ALT) levels only in the IDH2KO mice, suggesting early signs of impaired liver function (Fig. S2a–g). In accordance with the increased body weight gain and elevated body fat composition, a significant impairment in whole-body energy expenditure was observed in the HFD-fed IDH2KO mice but not in the LFD-fed IDH2KO mice (Fig. 1d, e). There were no differences observed in physical activity, food intake, or water consumption (Fig. S3a–c). These responses suggest that IDH2 deficiency drastically accelerates fat accumulation from a HFD, due at least in part to a reduction in the whole-body energy expenditure.

### HFD feeding impairs the iBAT function in the IDH2KO mice

**Fig. 2:**
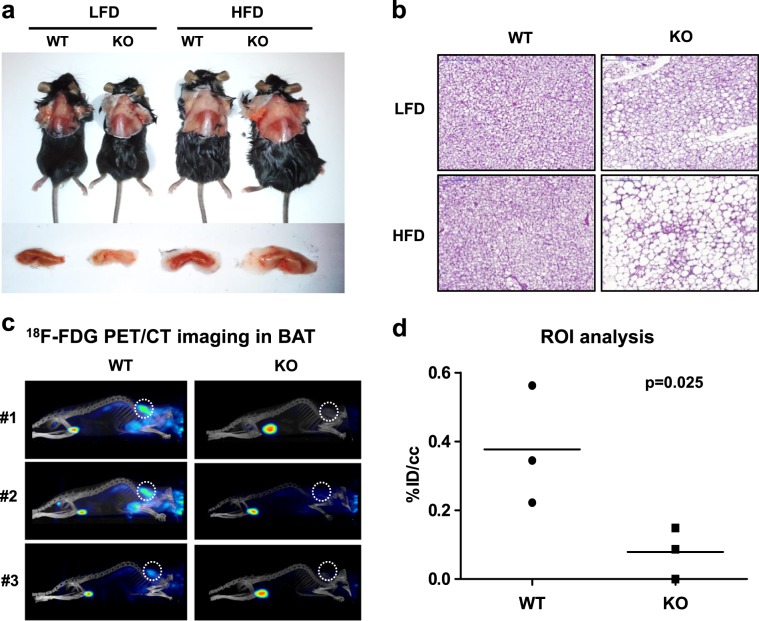
IDH2 deficiency leads to BAT whitening. **a** Representative images showing interscapular brown adipose tissue (iBAT) deposits (*n* = 6 per group). **b** Hematoxylin and eosin (H&E) staining of the iBAT section (5 μm) from the WT or IDH2KO mice exposed to a high-fat diet for 4 weeks. **c** PET/CT images showing the ^18^F-FDG uptake in the iBAT of the WT and IDH2KO groups as described in the “Materials and methods” (*n* = 5 per group). **d** Quantitative positron emission tomography–computed tomography (PET/CT) region of interest (ROI) analysis of the ^18^F-FDG uptake in the iBAT; ^18^F-FDG: 2-deoxy-2-[^18^F]-fluoro-d-glucose.

A tissue survey showed that IDH2 was expressed at high levels in the heart, kidney, and BAT (Fig. S4). Consistent with the role of IDH2 in regulating cardiac mitochondrial NADPH, a previous study showed that IDH2KO mice developed accelerated heart failure with impaired mitochondrial function, and were more susceptible to pathologic hypertrophy in a pressure-overload model^11^. Thus, we hypothesized that the BAT from the IDH2 mice might prevent the HFD-induced body weight gain and the associated metabolic consequences, including hepatic steatosis. Consistent with this model, visual inspection showed that the iBAT depots of the HFD-fed IDH2KO mice were enlarged and pale, suggesting an increase in the triglyceride accumulation and a decrease in the BAT energy expenditure (Fig. 2a). The histological comparisons were also consistent with the increased triglyceride accumulation in the iBAT depot from the IDH2KO group (Fig. 2b), indicating that the mutant iBAT is morphologically similar to the WAT tissue of the WT mice. Consistent with this hypothesis, the expression of BAT marker genes such as mitochondrial uncoupling protein-1 (*Ucp1)*, peroxisome proliferator-activated receptor gamma coactivator-1alpha (*Pgc-1α*), positive regulatory domain containing 16 (*Prdm16)*, and cell death-inducing DNA fragmentation factor α-like effector A (*Cidea)* were decreased (Fig. S5a), and the expression of signature WAT genes such as *Leptin* and *Adipoq* was significantly increased in the iBAT of the HFD-fed IDH2KO mice (Fig. S5b).

Given that BAT tissue is a major site for whole-body glucose uptake and metabolism^12^, we measured the uptake rate of ^18^F-FDG in the mice by PET/CT imaging. When we injected the IDH2KO mice with ^18^F-FDG, its uptake into the BAT was significantly reduced compared with that of the WT mice (Fig. 2c, d). This response is consistent with the reduced glucose uptake in the BAT from the IDH2KO group, and provides further evidence that iBAT function is significantly impaired in the HFD-fed IDH2KO mice.

### BAT dysfunction in the IDH2KO mice is due to mitochondrial dysfunction

**Fig. 3:**
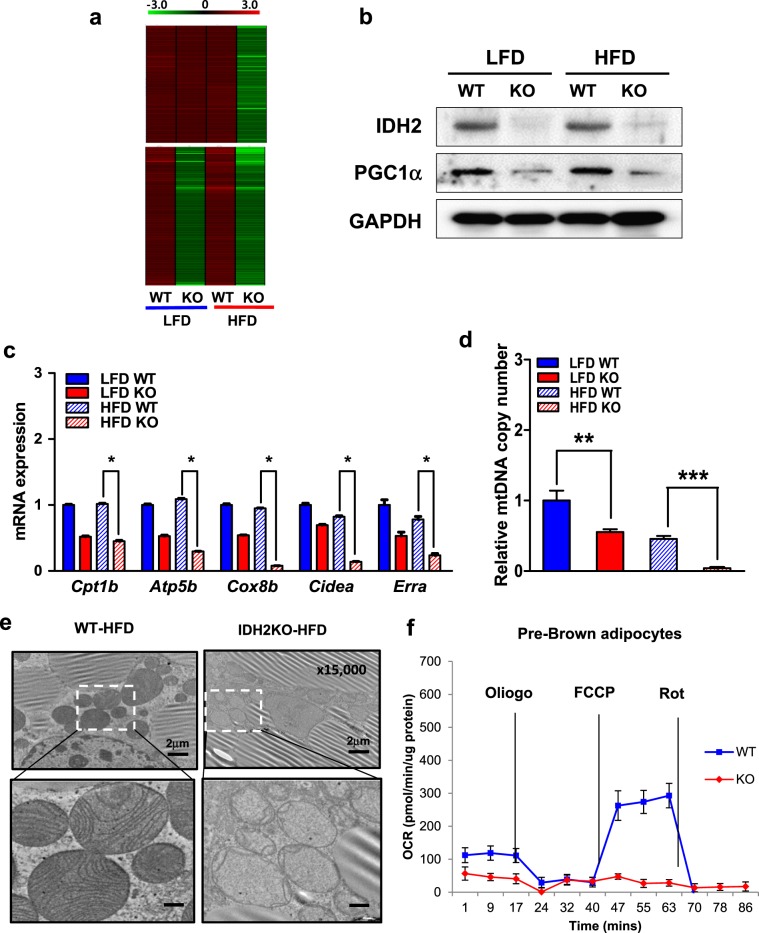
Depletion of IDH2 decreases the expression of mitochondrial-related genes and mitochondrial function in the BAT. Four-week-old WT and IDH2KO mice were fed a HFD for 4 weeks (*n* = 9 per group). **a** Heat map of the RNA-seq data representing genes downregulated in the IDH2KO group of HFD samples (top) or downregulated in both LFD and HFD (bottom). **b** Western blot analysis of IDH2 and PGC-1α from the iBAT. GAPDH was used as a loading control. **c** Expression of mitochondrial biogenesis genes in the iBAT of the WT and IDH2KO groups. **d** Relative mtDNA content expressed as a function of total genomic DNA (nDNA) in the WT and IDH2KO groups. **e** Transmission electron microscopy (TEM) showed multiple sphere-shaped mitochondria, which are characteristic of iBAT (*n* = 5). **f** Representative time course of the oxygen consumption rates (OCR) of primary brown adipocytes from the WT and IDH2KO mice. *p < 0.05, **p < 0.01, and ***p < 0.001 vs. HFD–WT or LFD–WT. TEM transmission electron microscopy.

Because IDH2 is a mitochondrial enzyme involved in redox balance, we hypothesized that the excess weight gain in the HFD-fed IDH2KO mice might result from mitochondrial dysfunction in the BAT. An RNA sequencing (RNA-seq) comparison of the gene expression profiles of the BATs in the WT and IDH2KO mice fed the LFD and HFD confirmed that many genes expressed at reduced levels in the IDH2KO mice are associated with mitochondrial function (Fig. S6a, b). These include genes downregulated only in the HFD-fed IDH2KO group (heat map in the top panel of Fig. 3a) or those decreased in both the LFD and HFD–IDH2KO groups (bottom panel in Fig. 3a). The reduced gene expression in the IDH2KO samples is at least partly due to the reduced activity of the PGC-1α protein, which was downregulated in the IDH2KO mice (Fig. 3b), and is a key transcriptional coregulator of many mitochondrial genes. In addition, the expression of estrogen-related receptor alpha (*Errα)*, a major transcriptional regulator of mitochondrial genes and a key DNA-binding partner for PGC-1α-mediated gene activation, was also significantly reduced in the IDH2KO mice (Fig. 3c) along with several known ERR–PGC-1 target genes analyzed directly by qPCR (Fig. 3c). The total levels of mitochondrial DNA were also decreased in the iBAT from the LFD-fed IDH2KO mice, and this parameter was reduced even further in the HFD group (Fig. 3d). Transmission electron microscopy (TEM) images also showed an aberrant mitochondrial structure with disrupted cristae in the iBAT from the HFD-fed IDH2KO group (Fig. 3e), but these defects did not differ between the LFD-fed WT and IDH2KO groups (Fig. S6c). A direct evaluation of mitochondrial respiration using the Seahorse system showed that the BAT from the IDH2KO group had a dramatic reduction in total oxygen consumption (Fig. 3f).

**Fig. 4:**
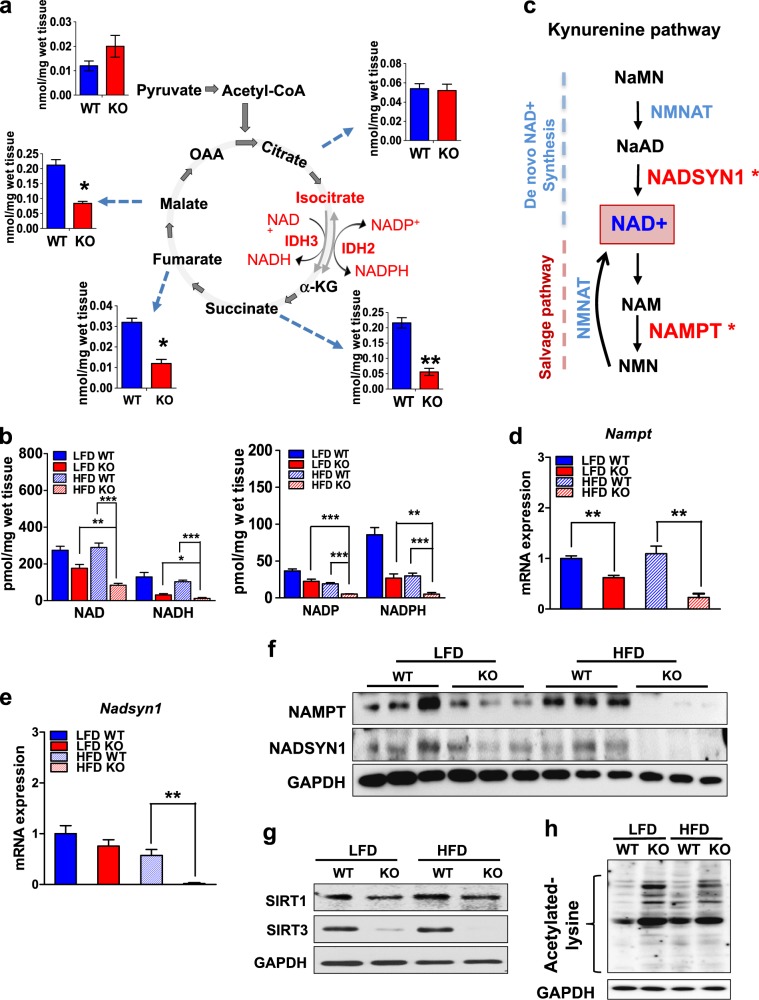
Nicotinamide levels are decreased in the iBAT of the IDH2KO mice. **a** Schematic model of the TCA cycle highlighting the IDH reaction in the TCA cycle. **b** The levels of total NAD^+^, NADH, NADP^+^, and NADPH were measured as described in the “Materials and methods” (*n* = 6 per group). **c** Scheme of the pathways highlighting NAD synthesis and recycling. **d–f** mRNA expression levels of *Nampt* and *Nads1* in the iBAT from the WT and IDH2KO mice, and proteins for NAMPT and NADSYN1 (proteins from nine mice from each group were prepared, and extracts from three individuals were pooled and treated as a separate sample for the gel). **g** Protein levels of SIRT1 and SIRT3 in the iBAT from the WT and IDH2KO mice. **h** Acetyl-lysine level of the whole proteins in the iBAT of the LFD and HFD–WT and KO mice. The proteins from six mice in each group were pooled and analyzed as individual samples in **g** and **h** (*n* = 6 per panel). *p < 0.05, **p < 0.01, and ***p < 0.001 vs. the LFD–WT mice, LFD–DH2KO mice, or HFD–WT mice. αKG α-ketoglutarate, OAA oxaloacetate, NaMN nicotinic acid mononucleotide, NaAD nicotinic acid adenine dinucleotide, NAD nicotinamide adenine dinucleotide, NAM nicotinamide, NMN nicotinamide mononucleotide, NMNAT NMN adenylyltransferase, NADSYN1 NAD synthetase 1, NAMPT nicotinamide phosphoribosyltransferase.

The IDH reaction within the TCA cycle is shown in Fig. 4a. When we measured the levels of TCA cycle intermediates and nicotinamide redox carriers, we noted that the TCA intermediates following IDH, as well as NAD^+^, NADH, NADP^+^, and NADPH, were significantly decreased in the BAT from the HFD-fed IDH2KO mice (Fig. 4a, b and Fig. S7). The RNA-seq data also suggested that the expression of nicotinamide adenine dinucleotide synthetase 1 (*Nadsyn1*) and nicotinamide phosphoribosyltransferase (*Nampt*) was decreased in the iBAT from the HFD-fed IDH2KO mice compared with the HFD-fed WT mice. These two enzymes are involved in producing NAD^+^ through the kynurenine and salvage pathways, respectively (Fig. 4c). Direct qPCR of the *Nadsyn1* and *Nampt* mRNA levels, and immunoblotting for the NAMPT protein, confirmed that they were reduced in the iBAT from the IDH2KO group (Fig. 4d–f).

The reduced levels of *Nadsyn1* and *Nampt* are consistent with the low levels of NAD^+^, NADH, NADP^+^, and NADPH. There was also a significant reduction in the levels of succinate with mild decreases in fumarate, malate, and citrate in the iBAT from the HFD-fed IDH2KO group compared with that from the HFD-fed WT group (Fig. 4a and Fig. S7). Taken together, the alterations in the metabolite and cofactor pools are consistent with a decrease in cellular redox control stemming from decreased IDH activity. In addition to its essential role as a soluble cofactor in electron transfer reactions, NAD^+^ is an allosteric regulator of the sirtuin family of deacetylases. In fact, PGC-1α and superoxide dismutase 2 mitochondria (SOD2) are directly deacetylated by sirtuin 1 (SIRT1)^13^ and sirtuin 3 (SIRT3)^14^, respectively, and both PGC-1α and SOD2 play important roles in mitochondrial function, as SOD2 is directly involved in ROS mitigation. Consistent with these previous observations, the expression of *Sirt1* was decreased, and that of *Sirt3* was dramatically reduced in the iBAT in both the LFD and HFD–IDH2KO samples (Fig. 4g). There were also significantly increased levels of overall protein acetylation in the IDH2KO mice, as measured by immunoblotting of total protein with an antibody that detects acetyl-modified lysine residues within proteins (Fig. 4h). The stability of the PGC-1α protein is increased directly by SIRT1-catalyzed deacetylation^15^, and the low PGC-1α protein levels were consistent with the reduced SIRT1 activity in the iBAT of the IDH2KO group (Fig. 3b).

### Loss of IDH2 leads to increased accumulation of ROS

**Fig. 5:**
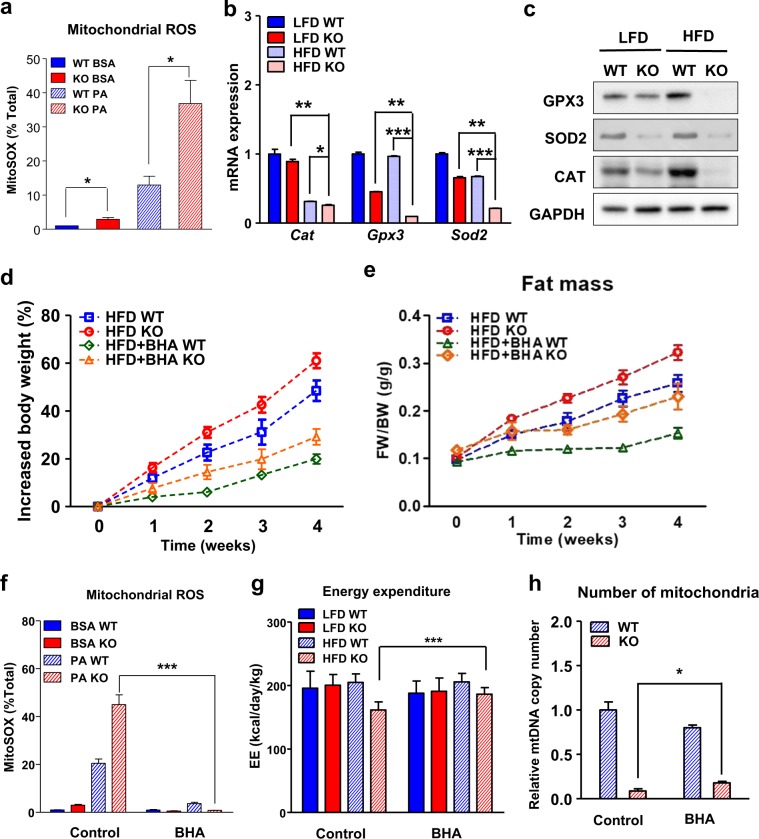
The effects of HFD on ROS production in the iBAT from the WT and IDH2KO mice, and the protective effect of an antioxidant against diet-induced obesity. **a** Primary brown adipocytes were cultured from the WT and IDH2KO mice (*n* = 3 per group) and treated with BSA or BSA plus palmitate, and the stained area was quantified with MitoSOX Red followed by FACS analysis. This experiment was repeated three times. **b**, **c** The mRNA and protein levels of key antioxidant genes/proteins in the iBAT from the WT and IDH2KO mice. **d**, **e** Growth curves and fat mass for control or butylated hydroxyanisole (BHA)-treated mice fed a LFD or HFD (*n* = 6 per group). **f** Primary brown adipocytes cultured from the WT and IDH2KO mice and treated with BSA or BSA plus palmitate, and the percentage of MitoSOX-positive cells stained with MitoSOX Red followed by FACS analysis (*n* = 3 per group). **g** Energy expenditure over a 24-h period of the WT and IDH2KO mice in the control or BHA-treated LFD and HFD groups. **h** Relative mitochondrial DNA content expressed as a function of total genomic DNA (mtDNA) in the iBAT from the control or BHA-treated HFD–IDH2KO mice. *p < 0.05, **p < 0.01, and ***p < 0.001 vs. the LFD–IDH2KO mice or HFD–WT mice.

To directly evaluate whether the ROS levels were affected, we treated the iBAT cells isolated from the WT and IDH2KO mice with superoxide-reactive MitoSOX Red dye, and FACS analysis showed a significant increase in the MitoSOX Red staining in the iBAT from IDH2KO mice, and the difference was maintained when the cells were treated with palmitate to increase the ROS production (Fig. 5a and Fig. S8). The mRNA and protein levels of catalase (CAT), SOD2, and GPX3, which are important regulators of cellular ROS, were also reduced in the BAT tissue from the IDH2KO mice in both the control and HFD groups (Fig. 5b, c). Taken together, our data suggest that loss of IDH2 results in defective ROS regulation in the BAT, and when the mice are subjected to metabolic stress from a HFD, they cannot limit the excess ROS accumulation, which leads to oxidative damage to the mitochondria. Thus, the excess caloric load cannot be purged through uncoupled heat production, and the carbon and energy are diverted to storage in the adipocyte fat droplets.

### Antioxidant supplementation reverses the metabolic imbalance and obesity in the HFD-fed IDH2KO mice

We hypothesized that inhibition of ROS accumulation would reverse mitochondrial dysfunction and limit the excessive weight gain phenotype in the HFD-fed IDH2KO mice. To test this hypothesis, we added the potent bioavailable antioxidant BHA^9^, which consists of a mixture of two isomeric organic compounds, 2-tert-butyl-4-hydroxyanisole and 3-tert-butyl-4-hydroxyanisole, to the HFD for the WT and IDH2KO mice. Interestingly, BHA supplementation significantly blunted the weight gain in both the WT and IDH2KO mice with no effect on food consumption (Fig. 5d and Fig. S9a–c). The reduction in weight gain was mirrored by a reduction in the total fat mass (Fig. 5e and Fig. S9d). The lean body mass was also normalized by the BHA treatment in both the WT and IDH2KO mice (Fig. S9e, f). BHA supplementation also reduced the levels of MitoSOX Red staining in the iBAT cells treated with palmitate (Fig. 5f and Fig. S9g) while increasing both energy expenditure and mitochondria number in the HFD-fed IDH2KO mice (Fig. 5g, h).

**Fig. 6:**
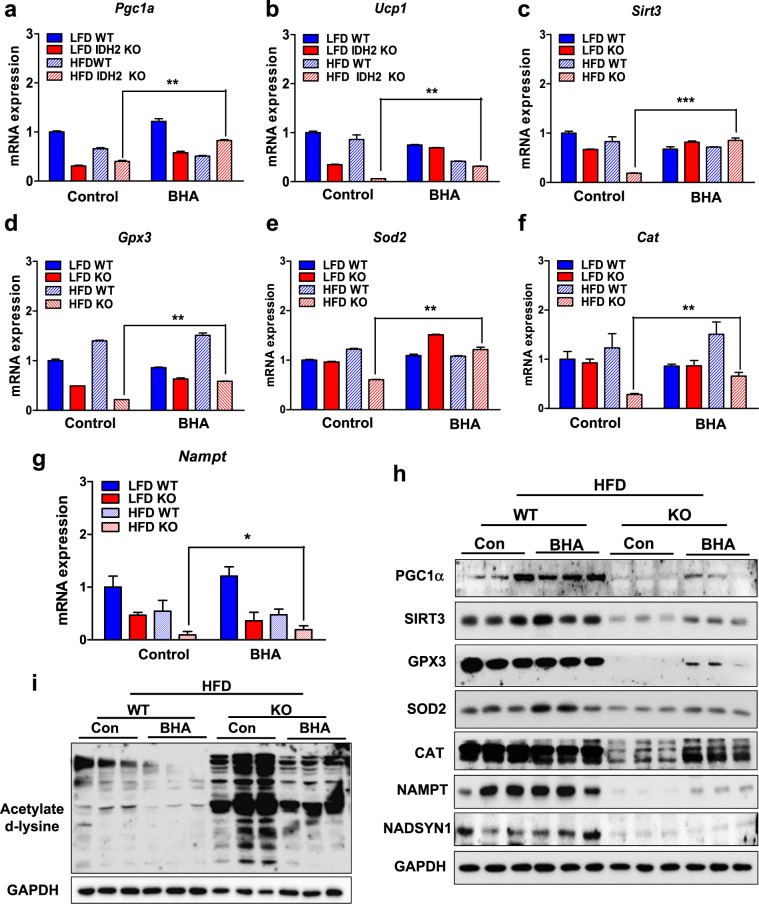
An antioxidant recovers mitochondrial function in the IDH2KO mice. Four-week-old WT and IDH2KO mice were fed a HFD, and were supplemented with BHA for 4 weeks (*n* = 6 per group). **a–g** qPCR analysis of the expression of the indicated genes. **h**, **i** The proteins from the BAT of the WT and IDH2KO mice were pooled from individuals and analyzed for the indicated proteins or for total acetylated lysine by immunoblotting. *p < 0.05, **p < 0.01, and ***p < 0.001 vs. the HFD–WT mice. PA palmitate, mtDNA mitochondrial DNA, EE energy expenditure.

To identify gene expression signatures that might help explain the BHA-mediated protection against the HFD-induced weight gain, we performed RNA-seq of the iBAT isolated from the WT and IDH2KO mice fed a HFD and supplemented with BHA. There were 1396 genes downregulated by 1.5-fold or more (*p* < 0.05) by the HFD in IDH2KO mice relative to WT mice (Fig. 3a and Fig. S10a), and 24 of these genes were significantly increased (*p* < 0.05) by BHA supplementation (Fig. S10b, c and Table S1). These included genes that contribute to mitochondrial function, energy expenditure, NAD regulation, and ROS resolution, such as *Pgc-1α, Ucp1, Sirt3, Gpx3, Sod2*, *Cat*, and *Nampt*, as well as *Nadsyn1* (Fig. 6a–g and Fig. S10d–k). In addition, the mitochondrial defects were partially reversed by the BHA treatment in the HFD-fed IDH2KO mice (Fig. S10l). The protein levels of PGC-1α, SIRT3, GPX3, CAT, and NAMPT showed at least partial recovery with the BHA supplementation (Fig. 6h). The increases in global protein acetylation levels were also reversed by the BHA treatment (Fig. 6i), consistent with the increase in SIRT protein levels. Taken together, these results suggest that the BHA-mediated protection against ROS resulted in at least partial recovery of mitochondrial function and body weight balance, suggesting that the major reason for the excess weight gain and mitochondrial dysfunction is due to elevated ROS downstream of the loss of IDH2.

## Discussion

WT mice fed a HFD showed increased uncoupled respiration in the BAT to defend against body weight gain from the excess caloric load^16^. An unavoidable metabolic consequence of elevated uncoupled respiration is increased ROS production, which needs to be limited to maintain mitochondrial homeostasis. In this study, we discovered that this adaptive response is disrupted in the IDH2KO mice. The HFD-mediated increase in mitochondrial activity overwhelmed the capacity of the mitochondria in the BAT of the HFD-fed mutant mice to limit ROS accumulation. This phenomenon results in severe oxidative damage to the mitochondrial enzyme systems involved in energy expenditure, and the mutant mice develop obesity at an accelerated rate relative to similarly fed WT mice. Notably, the metabolic parameters in Figs. 3–6 show that the LFD-fed IDH2KO mice also demonstrated signs of metabolic dysfunction, but these mice were not exposed to the excess caloric burden induced by exposure to the HFD challenge, and thus, their mitochondrial capacity was not subject to the overwhelming physiologic stress experienced by their HFD-fed littermates.

Other mitochondrial enzymes also produce NADPH to modulate redox balance, and are differentially regulated in response to different conditions. For example, glutamate dehydrogenase (GluDH) and mitochondrial NADP^+^ malic enzyme (ME3) are regulated by amino acid metabolism and directly affected by energy change through ATP, and nicotinamide nucleotide transhydrogenase (Nnt) is an inner mitochondrial membrane protein that uses energy from the proton gradient to generate NADPH^17^. However, Nnt is mutated and nonfunctional in wild-type C57BL/6J mice^18^, and GluDH and ME3 clearly did not compensate for the loss of IDH2 in the HFD-fed mice in our study. This hypothesis is likely because excess HFD calories are directly oxidized through the TCA cycle, where IDH2 is uniquely positioned to respond to the increased metabolite flow. Other pathways for NADPH production may be important in responding to situations in which ROS generation is directly linked to metabolite flow associated with these other enzyme systems, as described for mitochondrial glucose-6-phosphate dehydrogenase in response to glucose^19^.

IDH2 is one of three IDH isoforms that catalyze the oxidative decarboxylation of isocitrate to αKG. In the mitochondrion, this reaction is a key irreversible step of the TCA cycle, which is catalyzed predominantly by the IDH3 isoform, and is coupled with the capture of electrons by NAD^+^, forming NADH. Similar to IDH3, IDH2 is a mitochondrial matrix enzyme; however, it catalyzes a reversible reaction that utilizes NADP^+^ and NADPH as its redox pair (Fig. 4a). Mutated forms of IDH2 are associated with cancer, in which a mutant enzyme acquires the ability to produce the oncometabolite 2-oxoglutarate instead of αKG^20^. In contrast, little is known about the role of IDH2 in fundamental metabolism and metabolic adaptation. IDH2 is predicted to be involved in balancing mitochondrial NADP^+^ and NADPH levels as its major function. Mitochondrial NADPH is critically important to limit mitochondrial ROS accumulation when its activity is increased by providing the reducing power needed to convert the H_2_O_2_ produced by SOD2 from reactive O_2_^.–^ into H_2_O. In this scenario, the critical electron capture is coupled to the thioredoxin and glutathione redox cycles (Fig. S11).

Our study demonstrated that loss of IDH2 results in a major disruption of the mitochondrial adaptation to the metabolic stress of the HFD challenge in the BAT. There were reduced levels of both NAD^+^ and NADP^+^ in the HFD-fed IDH2KO mice, and this change was accompanied by decreased SIRT1 and SIRT3 activity, leading to increased global protein acetylation. PGC-1α and SOD2 are key mitochondrial proteins that are stabilized through deacetylation by Sirt1 and Sirt3, respectively^21,22^. Consistent with this mode of regulation, both PGC-1α and SOD2 decreased in the IDH2KO HFD-fed mice. The reduced levels of SOD2 would directly result in elevated O_2_^.–^, and the decline in PGC-1α would be coupled with reduced expression of many other key genes critical to mitochondrial function. Consistent with this critical role in mitochondrial gene expression, there was a severe decrease in thermogenic gene expression in a fat-specific PGC-1α-knockout mouse model^23^. In addition, the expression of *Errα* was significantly decreased in the IDH2KO HFD mice, and because ERRα is a major DNA-binding partner for PGC-1α, its reduction would lead to an even further decrease in the expression of key mitochondrial genes. These genes include *Sirt3* and *Sod2*, which are both activated by the concerted action of ERRα and PGC-1α^24–26^. In fact, prior studies have suggested that *Sirt3* is important for limiting oxidative damage^27^ through regulating IDH2 deacetylation^28,29^. The SIRT-dependent decline in the stability of SOD2 and PGC-1α mentioned above, combined with reduced gene expression, would lead to an even greater impairment of mitochondrial function. The RNA-seq profiling and direct qPCR and immunoblotting results also revealed decreased expression of the *Nadsyn1* and *Nampt* genes, which are involved in NAD biosynthesis and regeneration, respectively (Fig. 4c). This phenomenon likely explains the reduction in NAD^+^ and NADP^+^.

We predicted that the increased weight gain in the HFD-fed IDH2 mice might be secondary to the ROS-mediated damage to the mitochondria in the BAT. Interestingly, dietary supplementation of the antioxidant BHA with the HFD limited the excess weight gain, reversed the elevated levels of global protein acetylation, decreased mitochondrial ROS, and significantly improved the mitochondrial function in response to the loss of IDH2. These changes were accompanied by a rebound in the expression of *Pgc-1α*, *Ucp1*, and *Sirt3* with a modest recovery in *Nampt*, *Nadsyn1*, and *Sod2*. These responses provide strong support for the hypothesis that the HFD-induced weight gain in IDH2KO mice was due to ROS-mediated mitochondrial damage in the BAT.

The levels of TCA cycle intermediates downstream of IDH2—succinate, fumarate, and malate—were also decreased in the BAT from the IDH2KO animals (on both regular chow and HFD). There was no change in the level of citrate in the HFD-fed mice. For the majority of samples, the levels of αKG fell below our limit of quantitation. Interestingly, succinate promotes H_2_O_2_ production in the presence of NAD^+^-dependent substrates that feed into complex 1 of the ET chain^30^. Hence, the reduced level of succinate may reflect a partial compensatory-protective mechanism to downregulate H_2_O_2_ production in the face of both IDH2 deficiency and HFD challenge. Notably, although IDH2 is not believed to play a significant role in carbon flux through the TCA cycle under normal conditions, the decreased levels of succinate, fumarate, and malate suggest that IDH2 activity may contribute to bulk TCA cycle flux when the mitochondrial redox balance is perturbed. Moreover, the reduced TCA flux of citrate through succinate could also result in more citrate diverted to the cytoplasm for lipid biosynthesis, which could contribute to the rapid weight gain in the IDH2KO mice fed the HFD.

Carnitine palmitoyl transferase 1β (CPT1β) is responsible for transferring fatty acyl-CoAs from the cytoplasm to the mitochondria, which is the rate-limiting step for fatty acid oxidation. Because CPT1β levels are reduced in the iBAT from the HFD-fed IDH2KO mice, the excess calories would be diverted away from mitochondrial oxidation and shuttled to excess fat storage, which would also contribute to the increased susceptibility of the IDH2KO mouse to HFD-induced obesity.

In addition to their roles in the TCA cycle itself, key intermediates have fundamental roles in regulating enzymes involved in diverse cellular processes. For example, αKG is a cosubstrate for a nonheme iron-dependent class of dioxygenase enzymes that demethylate histones and DNA^31^. Importantly, these enzymes are also inhibited by succinate and fumarate^32^. Because the loss of IDH2 resulted in a decrease in succinate and fumarate and possibly αKG itself, it is likely that at least part of the effects we observed on gene expression are mediated through altered methylation of DNA or histones as a consequence of the decreased levels of TCA cycle intermediates. However, because most of the key mitochondrial genes we have highlighted are directly regulated by PGC-1α and/or ERRα, which would also alter gene expression through changes to the chromatin structure, the contributions from altered TCA cycle intermediates to the broader mechanism presented here will require more precise studies that selectively target this pathway.

Our results are consistent with a role for IDH2 in limiting ROS-mediated damage to mitochondrial function in the BAT. A supporting paper on the role of IDH2 in mitochondrial ROS regulation in the liver was also recently published^33^. In this report, IDH2KO mice fed a HFD for 16 weeks gained excess weight and developed hepatic steatosis, but the mechanism driving the excess weight gain was not explored, and energy expenditure was not evaluated. The HFD–IDH2KO mice in our study also developed more severe hepatic steatosis than the WT mice, and this phenomenon was evident after only 4 weeks on the HFD. There was also an increase in the serum ALT levels in our mice (Fig. S2f). These responses are consistent with the compromised liver function after 4 weeks of HFD, but there was no decrease in the liver mitochondrial DNA at this time point when the mitochondrial function in the BAT was dramatically reduced. In addition, IDH2 expression in the liver is minor relative to that in the BAT (Fig. S4), and thus, the liver steatosis at 4 weeks of HFD likely develops at least partially as a secondary effect from defective ROS regulation in the BAT that leads to a deficit in whole-body expenditure, resulting in increased levels of excess fat deposition in the liver and compromised liver function by 16 weeks of HFD.

One coauthor of this study (J.W.P) was part of a previous study that followed the weight gain in HFD-fed WT and IDH2KO mice starting at 6 months of age^34^. In contrast to the current studies and the publication mentioned in the previous paragraph, the study starting with 6-month-old mice reported that the IDH2KO mice gained much less weight than the WT mice on both LFD- and HFD-feeding regimens. The reason our study showed opposite results when performed on much younger mice is likely because the chronic loss of IDH2 results in adverse metabolic deterioration, and by 6 months of age, the mice respond differently when challenged with HFD. In support of this explanation, another earlier publication from the same group showed an age-related decline in cardiac function in IDH2KO mice^11^.

In summary, we propose that deletion of *Idh2* increases the metabolic stress from HFD. The ensuing elevation of ROS overwhelms the capacity of the BAT mitochondrial homeostatic system to reduce the resulting oxidative damage that cripples critical mitochondrial enzyme systems, leading to a severe decline in BAT function. Importantly, the effects can be partially reversed by supplementation with a broadly bioavailable antioxidant to the diet. These findings support the hypothesis that a major detrimental effect from the loss of IDH2 is the accumulation of ROS. Many studies have suggested that dietary supplementation with antioxidants may provide various health benefits in humans^35^, and oxidative stress is identified as a major complication in human obesity^36^. Thus, our studies may help elucidate the mechanism for at least some of the health benefits of antioxidant supplementation in obese humans.

## Supplementary information

Supmementary materials
