# Correction: Isocitrate dehydrogenase 2 protects mice from high-fat diet-induced metabolic stress by limiting oxidative damage to the mitochondria from brown adipose tissue

**DOI:** 10.1038/s12276-020-0451-8

**Published:** 2020-06-15

**Authors:** Jae-Ho Lee, Younghoon Go, Do-Young Kim, Sun Hee Lee, Ok-Hee Kim, Yong Hyun Jeon, Taeg Kyu Kwon, Jae-Hoon Bae, Dae-Kyu Song, Im Joo Rhyu, In-Kyu Lee, Minho Shong, Byung-Chul Oh, Christopher Petucci, Jeen-Woo Park, Timothy F. Osborne, Seung-Soon Im

**Affiliations:** 1 Department of Physiology, Keimyung University School of Medicine, Daegu, 42601 Republic of Korea; 2 Department of Internal Medicine, School of Medicine Kyungpook National University, Kyungpook National University Hospital, Daegu, 41944 Republic of Korea; 3 Leading-Edge Research Center for Drug Discovery and Development for Diabetes and Metabolic Disease, Kyungpook National University Hospital, Daegu, 41404 South Korea; 4 Korean Medicine Application Center, Korea Institute of Oriental Medicine, Daegu, 41062 Republic of Korea; 5 Department of Physiology, Lee Gil Ya Cancer and Diabetes Institute, Gachon University School of Medicine, Younsoo-gu, Inchon 21999 Republic of Korea; 6 Laboratory Animal Center, Daegu-Gyeongbuk Medical Innovation Foundation, Daegu, 41061 Republic of Korea; 7 Department of Immunology, Keimyung University School of Medicine, Daegu, 42601 Republic of Korea; 8 Department of Anatomy, Korea University College of Medicine, Seoul, 02841 Republic of Korea; 9 Research Center for Endocrinology and Metabolism, Chungnam National University Hospital (CNUH), 282 Munhwaro, Daejeon, 35015 Republic of Korea; 10 Center for Metabolic Origins of Disease, Sanford Burnham Prebys Medical Discovery Institute, Orlando, FL 32827 USA; 11 Cardiovascular Institute and Department of Medicine, Perelman School of Medicine at the University of Pennsylvania, Philadelphia, PA 19104 USA; 12 School of Life Sciences and Biotechnology, College of Natural Science, Kyungpook National University, Daegu, 41566 Republic of Korea; 13 Institute for Fundamental Biomedical Research, Department of Medicine and Biological Chemistry, Johns Hopkins University School of Medicine, St. Petersburg, FL 33701 USA

**Keywords:** Obesity, Transcriptional regulatory elements

Correction to: *Experimental & Molecular Medicine* 10.1038/s12276-020-0379-z published online 3 February 2020

After the online publication of this article, the authors noticed an error in the Acknowledgments section.

The correct statement of this article should read as follows.

The authors wish to change grant number HR18C0012 to HI14C1324. Please add “This study was funded by the Ministry of Health & Welfare, Republic of Korea (HI14C1324) to S.S.I.” in the Acknowledgments.

The authors apologize for any inconvenience caused.

