# Supplementary material for: Isocitrate dehydrogenase 2 protects mice from high-fat diet-induced metabolic stress by limiting oxidative damage to the mitochondria from brown adipose tissue
Source: Exp Mol Med. 2020 Feb 3;52(2):238–52. doi: 10.1038/s12276-020-0379-z (PMC7062825; doi:10.1038/s12276-020-0379-z)
Supplement: Supplementary file 1 — Supmementary materials [file 12276_2020_379_MOESM1_ESM.docx]

**SUPPLEMENTARY MATERIALS**

**Histological analysis**

BAT tissues were fixed in 4% paraformaldehyde and subsequently embedded in paraffin (4–5 μm sections) for haematoxylin and eosin (H&E) staining. H&E staining was performed using a standard protocol and stained sections were imaged with the Zeiss Axio Imager Z1 microscope.

**Quantitative RT-PCR**

Total RNA was prepared from tissue of WT and IDH2KO mice by the Trizol procedure (Invitrogen, Carlsbad, CA, USA) and cDNA was synthesized using cDNA superscript kit (Bio-Rad, Richmond, CA, USA) to use for qPCR with CFX96 Bio-Rad qPCR machine (Bio-Rad). mRNA levels were normalized for expression of ribosomal protein L32 mRNA as control and calculated by the comparative threshold cycle method. The primer sequences are listed in Table S2.

**TG and cholesterol measurement in serum**

Serum lipids (TG, cholesterol, HDL, LDL, and NEFA) and liver toxicity (AST and ALT) concentrations were measured by a Beckman Coulter AU480 automatic biochemistry analysis system (Model AU-480, Beckman Coulter, Krefeld, Germany).

**Supplementary figures**

**
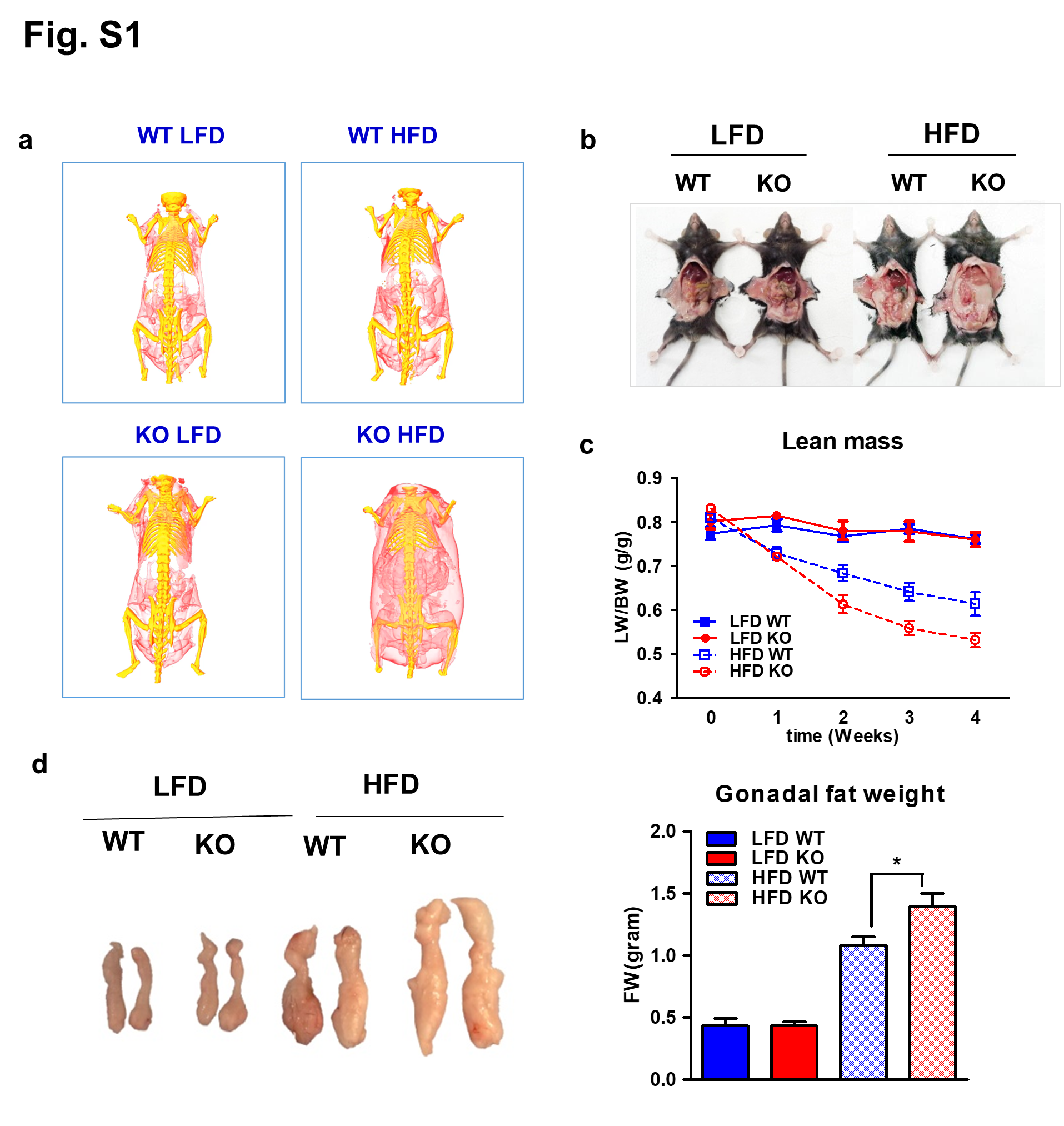
**

**Fig. S1 Increase of fat accumulation by HFD in IDH2KO mice compared to WT mice.**  **Increase of fat accumulation by HFD in IDH2KO mice compared to WT mice.**  **a** CT images of representative mice on LFD and HFD for 4 weeks (n = 6 per each group). Red color indicates fat mass. **b** Photographs of representative mice abdominal fat of each group (LFD-WT, LFD-IDH2KO, HFD-WT, and HFD-IDH2KO mice) after 4 weeks. **c** Time course of changes in lean mass. **d** Representative images and fat weight graph showing intra-abdominal fat deposits of **b**. **p* < 0.05 vs. HFD-WT mice.

**
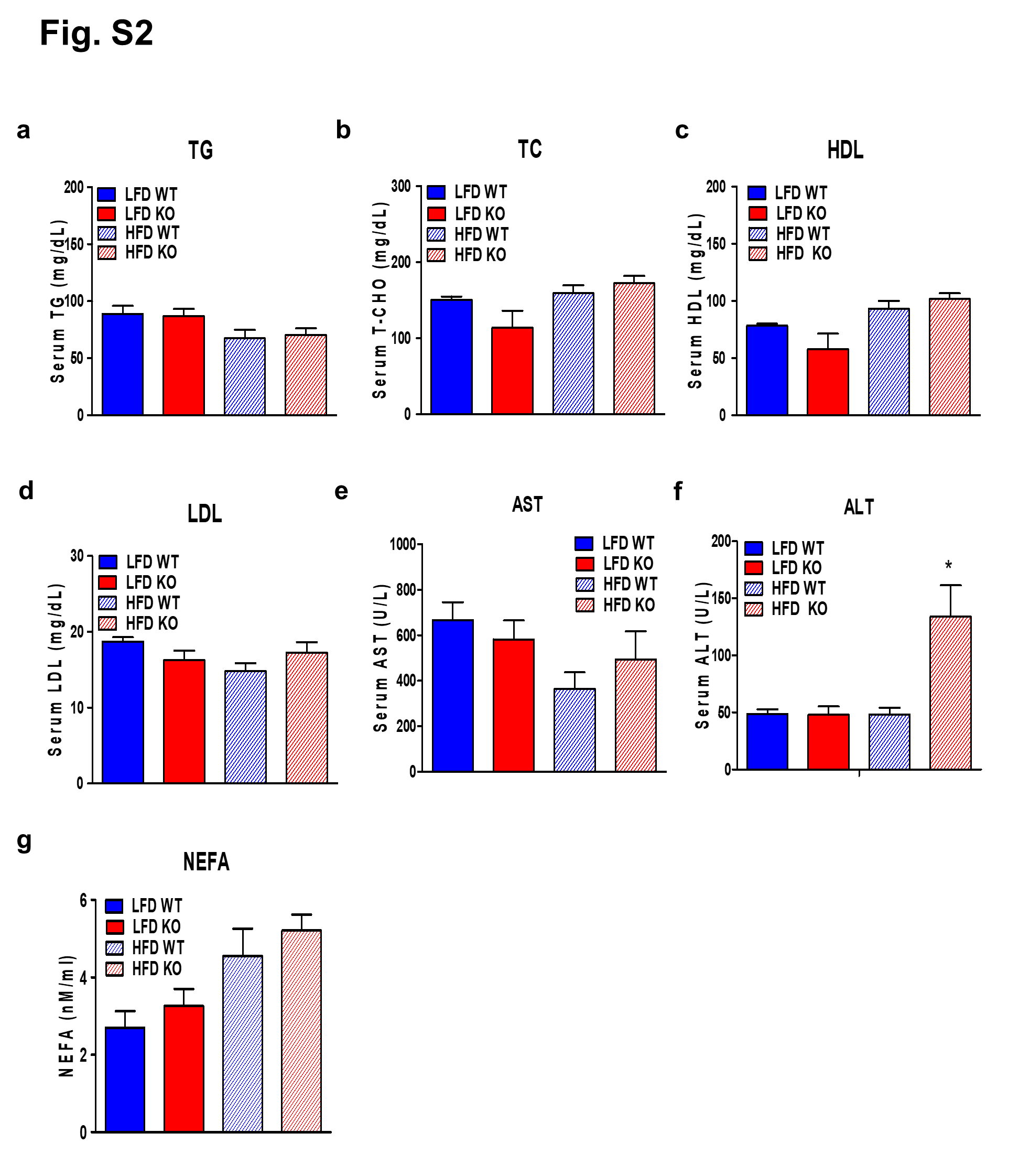
**

**Fig. S2 Effect on serum lipid profiles on an LFD or HFD.** **a-g** Serum lipid and liver toxicity marker profiling analysis of WT and IDH2KO mice after LFD and HFD. **p* < 0.05 vs. HFD-WT mice (n = 6 per each group). TG: Total triglyceride, TC: Total cholesterol, HDL: High-density lipoprotein, LDL: Low-density lipoprotein, AST: Aspartate transaminase, ALT: Alanine transaminase, NEFA: Non-esterified fatty acids.

**
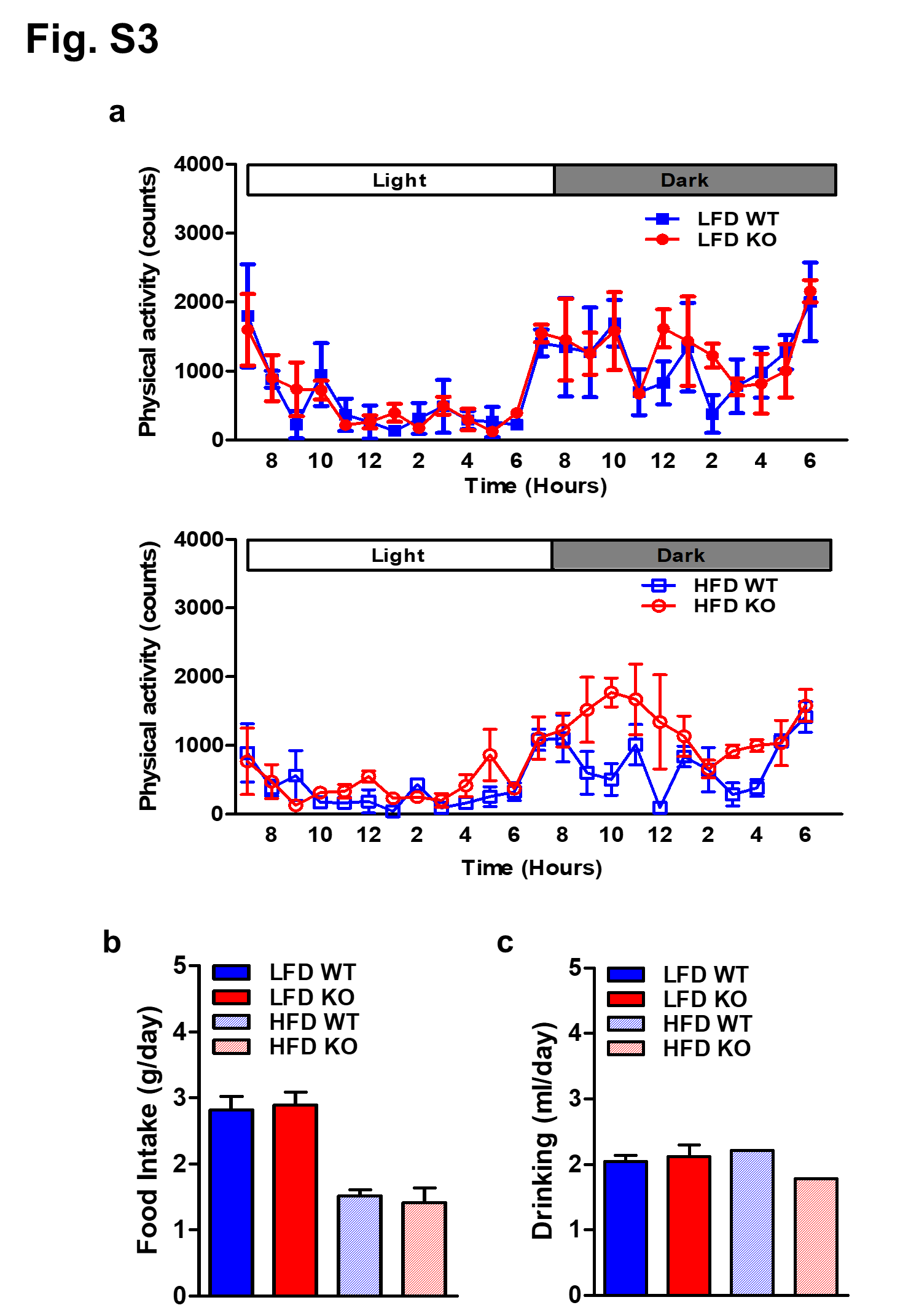
**

**Fig. S3 period**, **WT and IDH2KO mice fed with LFD and HFD.** **a** Physical activity during 12 h light and dark cycles recorded at the second day after acclimatization in WT and IDH2KO mice fed with LFD and HFD. **b, c** Average amount of food and water intake over a 24 h (n = 6 per each group).


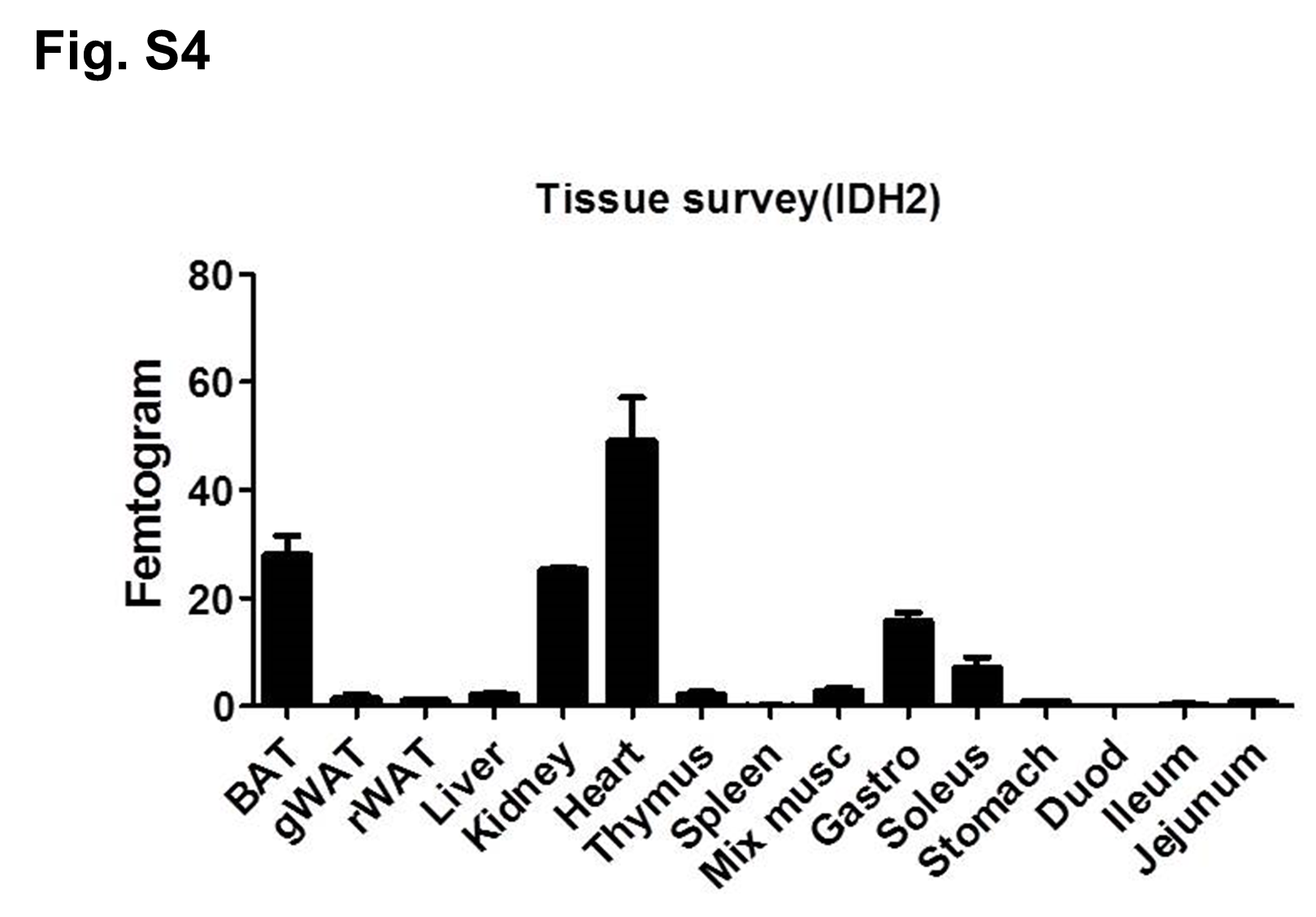


**Fig. S4 Tissue survey of IDH2 gene expression level.** Total RNA from the indicated tissues were used for qPCR with an internal control DNA standard for quantitation. Values are presented as fg of RNA/ mg total RNA. Data are from pooled RNA samples from WT (n = 5). BAT: brown adipose tissue, gWAT: gonadal white adipose tissue, rWAT: retroperitoneal white adipose tissue, Duod: duodenum.

**
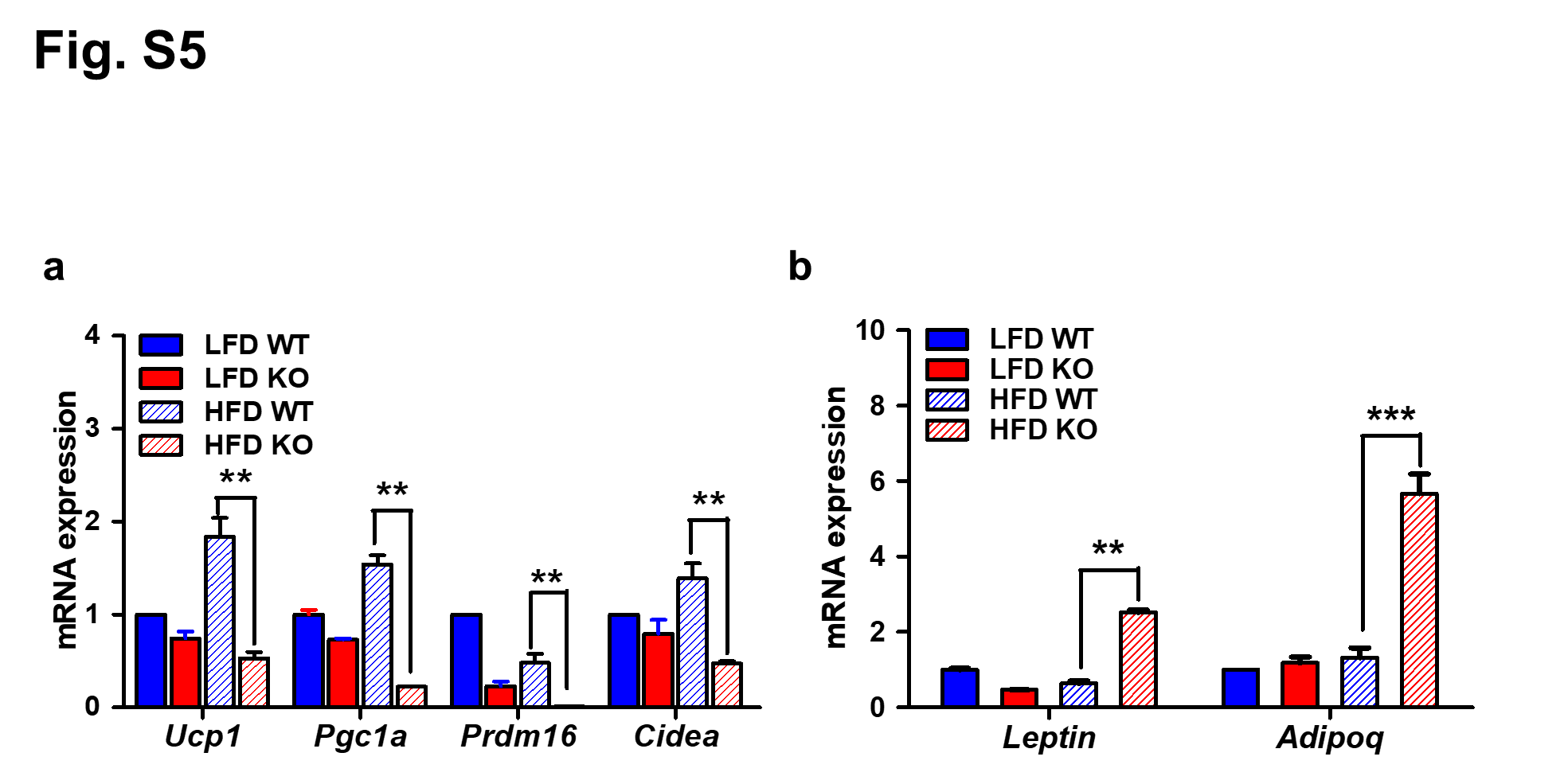
**

**Fig. S5 Gene expression levels in iBAT of HFD-fed mice.** **a** Expression levels of iBAT marker genes in iBAT were assessed by qPCR. **b** mRNA levels of genes related to WAT in iBAT. Total mRNAs were collected and were analyzed by qPCR with indicated primers. ***p* < 0.01 and ****p* < 0.001 vs. HFD-WT mice. n = 6 per each group.

**
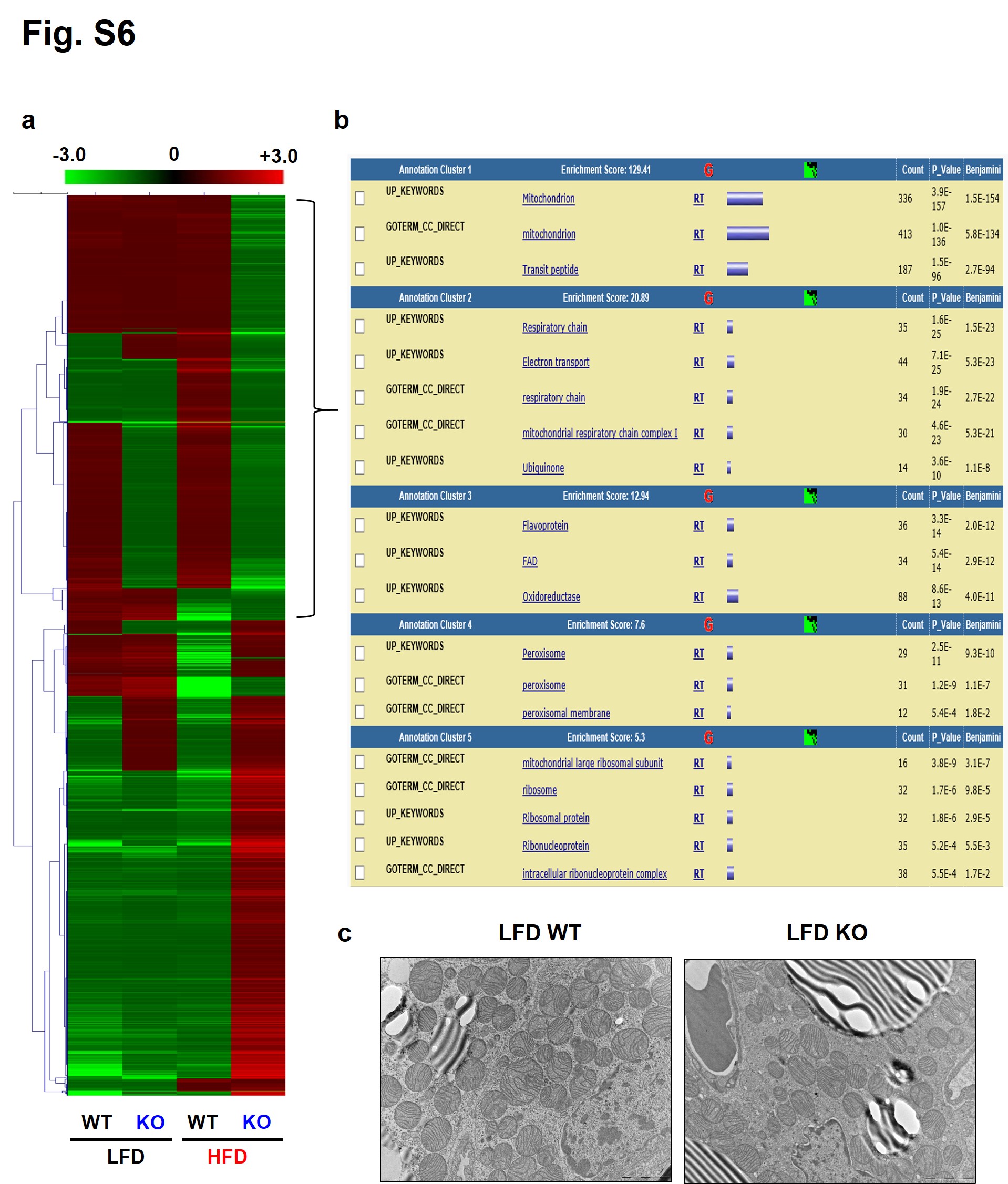
**

**Fig. S6 Heatmap and gene ontology analysis from RNA-Seq data.** **a** Heatmap representation of gene expression from iBAT of WT and IDH2KO mice fed LFD and HFD. **b** Gene ontology (GO) result in Biological Process (BP) categories. Decreased genes (1396 genes) in HFD KO vs HFD WT from the RNA-seq were imported into DAVID. GO term analysis was performed to identify biological processes enriched among decreased genes. (n = 6 per each group). **c** Transmission electron microscopy (TEM) showed multiple sphere-shaped mitochondria, which is characteristic of iBAT (n = 5).


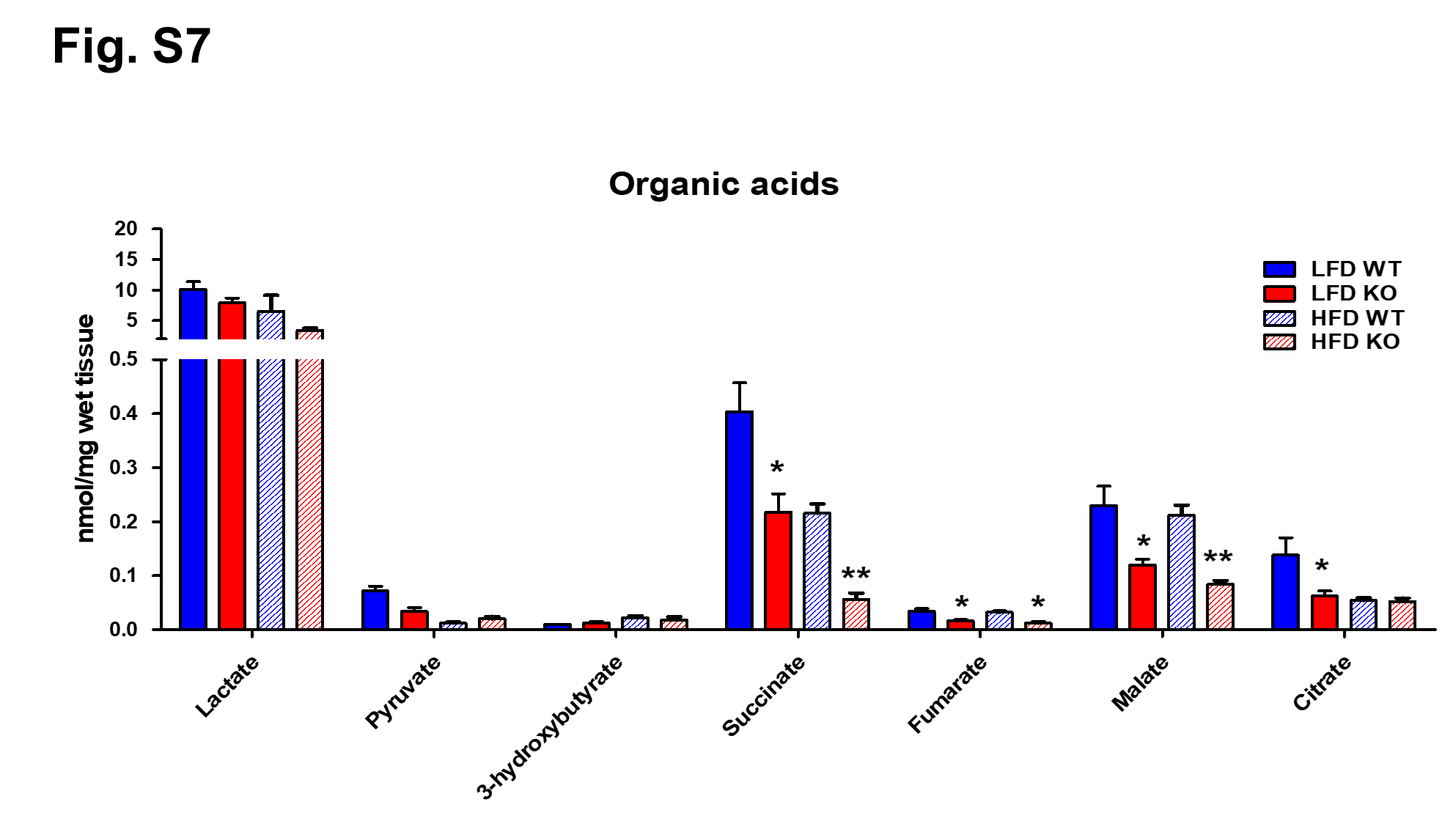


**Fig. S7 Organic acids level.** Organic acids levels were measured as detailed in materials and methods. **p* < 0.05 vs. LFD-WT mice or HFD-WT mice, and ***p* < 0.001 vs. HFD-fed WT mice.


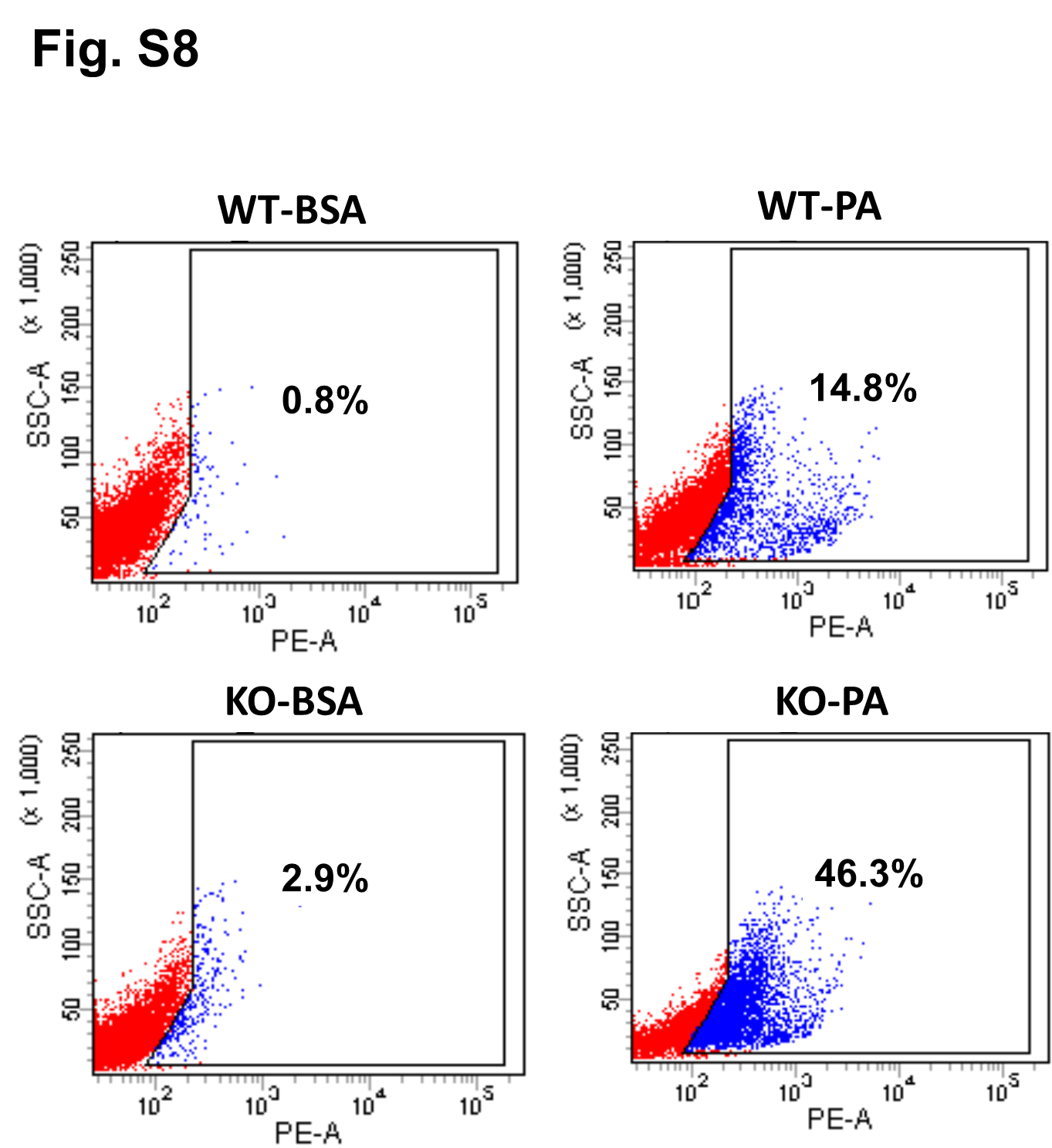


**Fig. S8 FACS analysis.** Primary brown adipocytes were isolated from WT and IDH2KO mice (n = 3 per each group) and treated with BSA or BSA plus palmitate and stained with MitoSOX Red followed by FACS analysis.

**
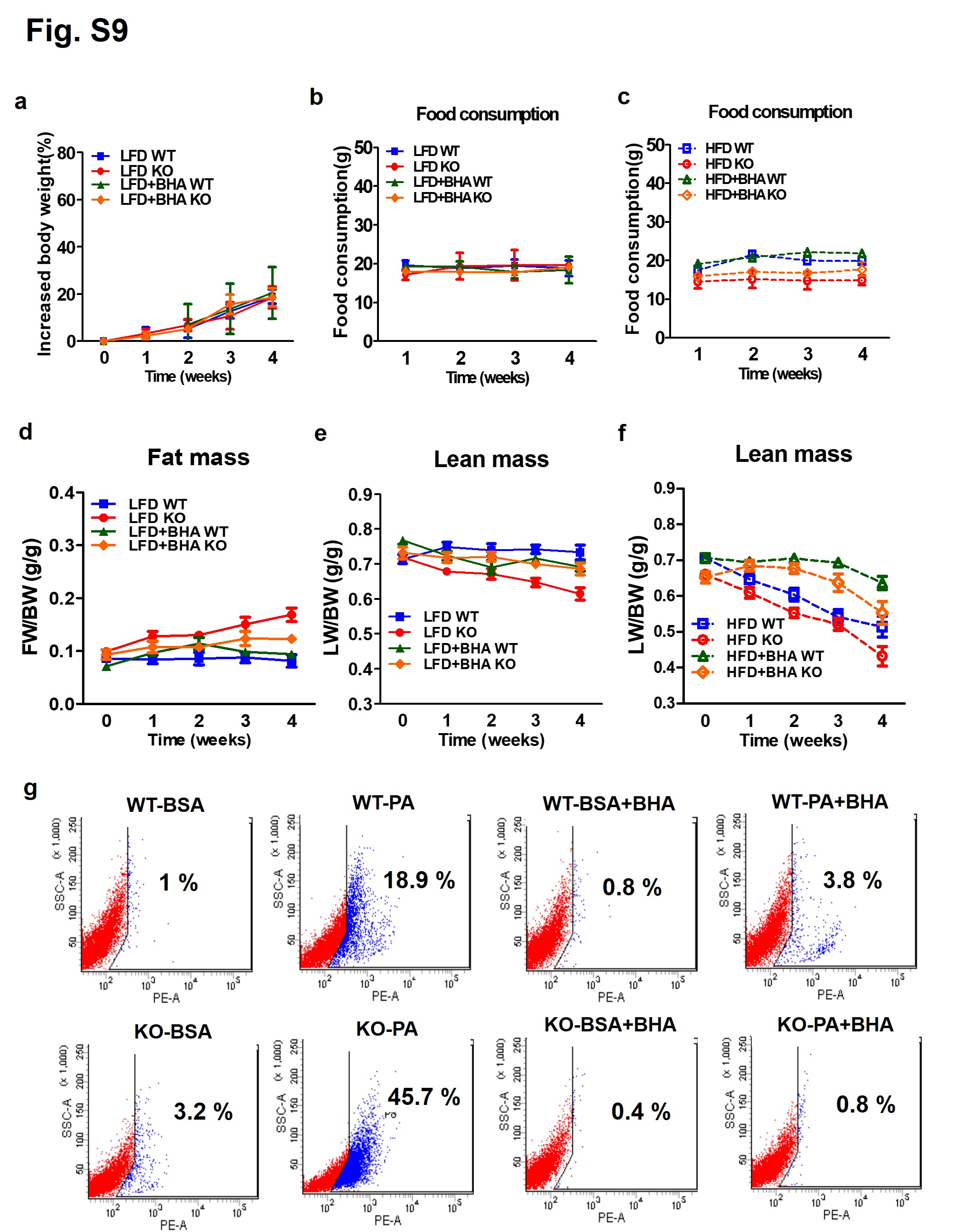
**

**Fig. S9 Energy expenditures and body composition in response to BHA treatment in WT and IDH2KO mice.** **a** Increased body weight. **b** Food consumption in LFD or LFD plus BHA-fed WT and IDH2KO mice groups. **c** Food consumption in HFD or HFD plus BHA-fed WT and IDH2KO mice. **d-f** Fat mass data in LFD and LFD plus BHA-fed WT and IDH2KO mice. Lean mass data in LFD or HFD with control or BHA-fed WT and IDH2KO mice (n = 6 per each group). **g** Primary brown adipocytes were isolated from WT and IDH2KO mice and treated with BSA or BSA plus palmitate and stained with MitoSOX Red followed by FACS analysis.


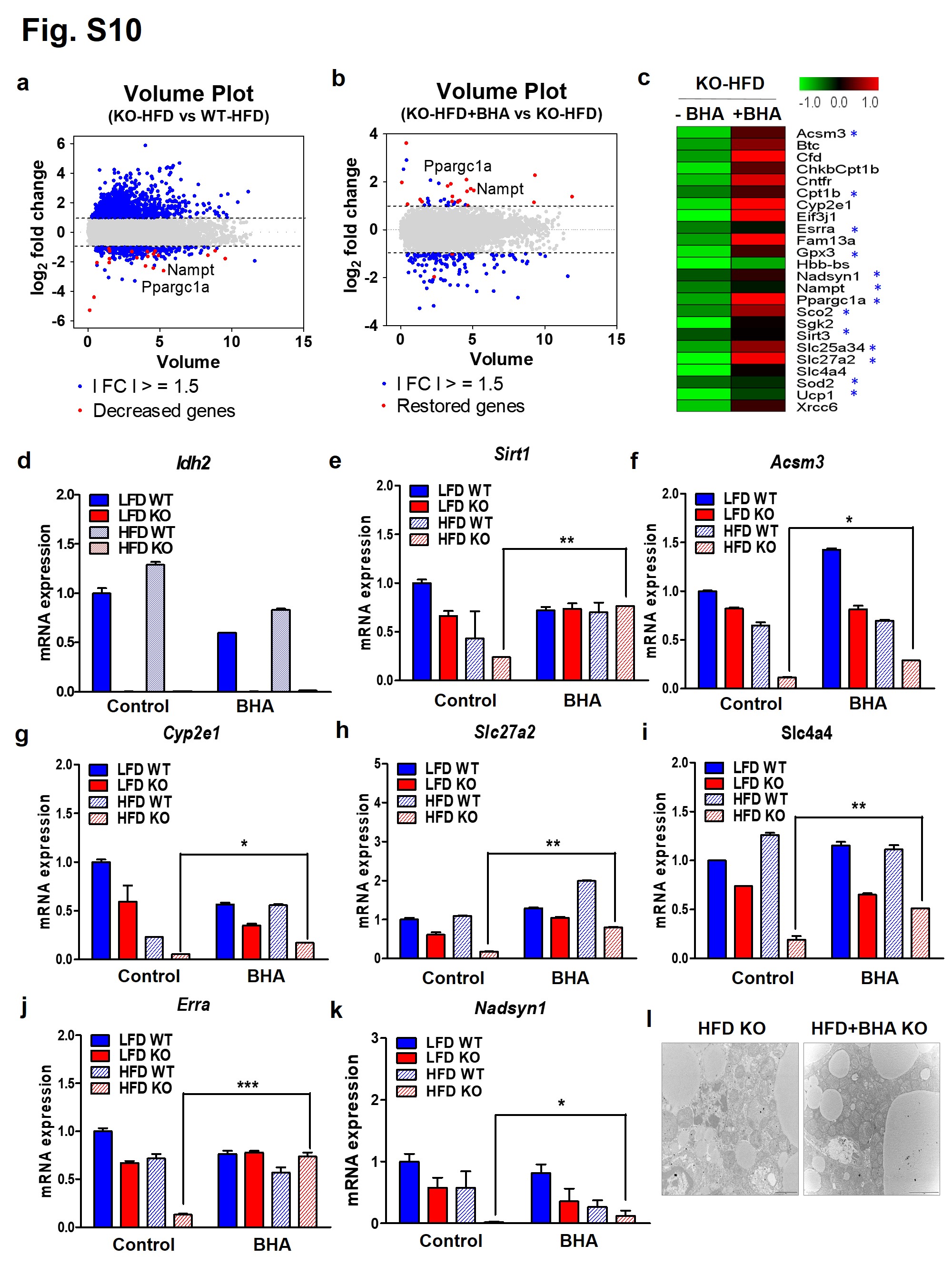


**Fig. S10 Gene expression by BHA in BAT. Gene expression by BHA in BAT.** RNA-seq was performed as described in methods. WT and IDH2KO mice were fed with HFD plus BHA for 4 weeks. **a, b** Scatterplot of the gene expression ratio in control versus BHA-treated LFD or HFD-IDH2KO mice. **c** Heatmap representation of gene expression differences between control and BHA-treated HFD-IDH2KO mice (n = 9 per each group) as determined by RNA sequencing of iBAT. RNAs were isolated from iBAT and gene expression levels for several genes listed in Table S1 were measured by qPCR. **d-k** *Idh2*, *Sirt1*, *Acsm3*, *Cyp2e1*, *Slc27a2*, *Slc4a4*, *Erra*, and *Nadsyn1*.  **l** TEM showed multiple sphere-shaped mitochondria, which is characteristic of iBAT (n = 5). **p* < 0.05, ***p* < 0.01, and ****p* < 0.001 vs. HFD-fed WT mice.

**
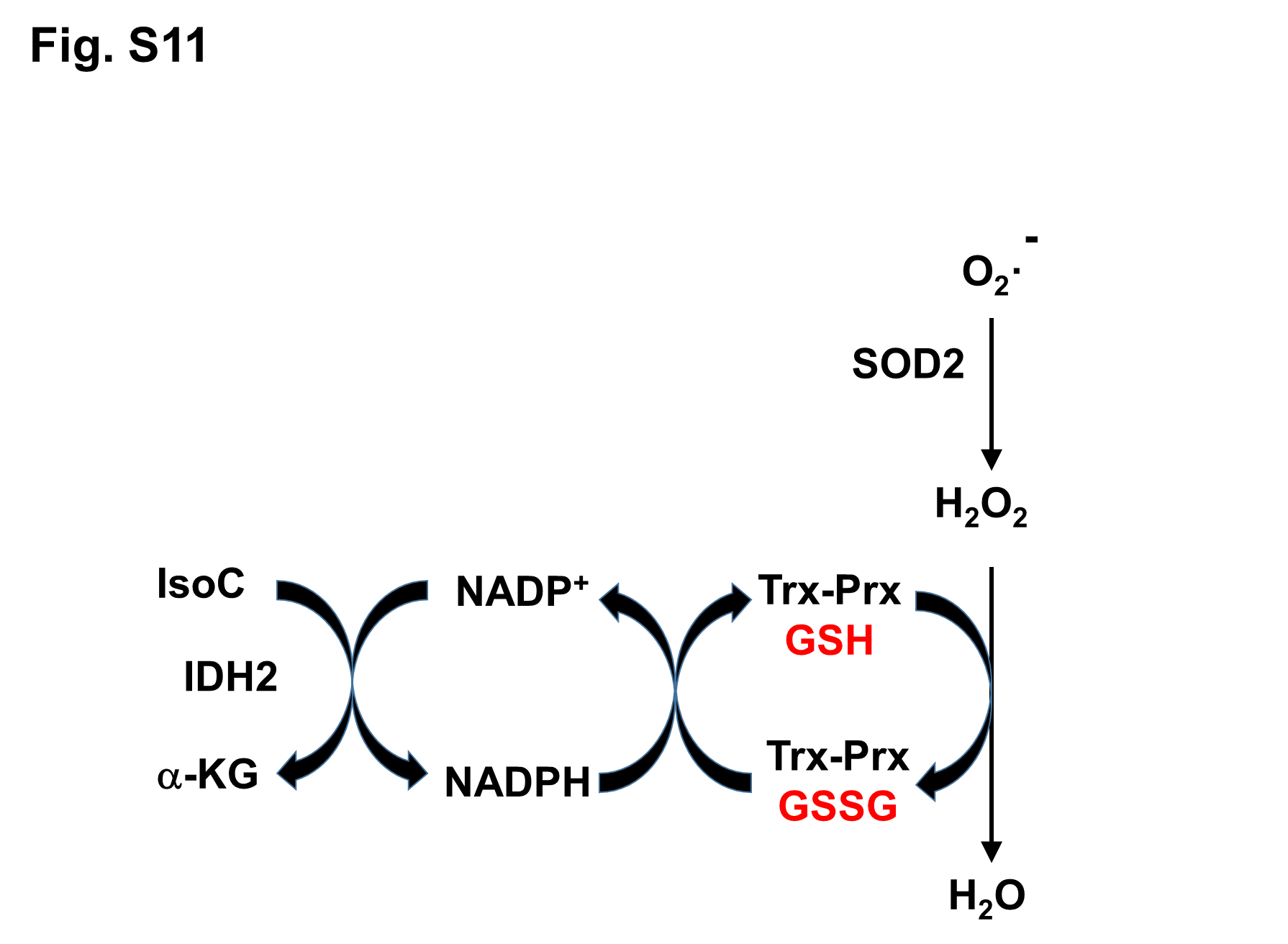
**

**Fig. S11 Scheme of electron flow through thioredoxin and glutathione redox cycles. Table S1 Genes decreased in HFD-fed KO BAT that were recovered by BHA supplementation.**

| Number | Transcript_ID | Gene_  Symbol | Gene_Description | HFD_KO/HFD_WT.fc | HFD_BHA_KO/HFD_KO.fc |
| --- | --- | --- | --- | --- | --- |
| 1 | NM_016870, NM_212442, NM_212441 | Acsm3 | acyl-CoA synthetase medium-chain family member 3 | -3.104 | 2.226 |
| 2 | NM_007568 | Btc | betacellulin, epidermal growth factor family member | -2.413 | 2.246 |
| 3 | NM_001291915,NM_013459 | Cfd | complement factor D isoform 2 precursor | -2.37 | 4.854 |
| 4 | NR_004843 | ChkbCpt1b | Chkb-Cpt1b readthrough transcript (NMD candidate) | -3.458 | 2.398 |
| 5 | NM_001136056,NM_016673,NM_001146080 | Cntfr | ciliary neurotrophic factor receptor | -2.777 | 3.041 |
| 6 | NM_009948 | Cpt1b | carnitine palmitoyltransferase 1b, muscle | -1.98 | 1.713 |
| 7 | NM_021282 | Cyp2e1 | cytochrome P450, family 2, subfamily e, polypeptide 1 | -3.311 | 3.774 |
| 8 | NM_144545 | Eif3j1 | eukaryotic translation initiation factor 3, subunit J1 | -20.984 | 12.248 |
| 9 | NM_007953 | Esrra | estrogen related receptor, alpha | -1.995 | 1.344 |
| 10 | NM_153574 | Fam13a | family with sequence similarity 13, member A | -2.4 | 3.271 |
| 11 | NM_008161 | Gpx3 | glutathione peroxidase 3 | -3.44 | 2.208 |
| 12 | NM_001201391 | Hbb-bs | hemoglobin, beta adult s chain | -38.779 | 3.934 |
| 13 | NM_030221 | Nadsyn1 | NAD synthetase 1 | -1.593412 | 1.438193 |
| 14 | NM_021524 | Nampt | nicotinamide phosphoribosyltransferase | -2.023445 | 1.335968 |
| 15 | NR_027710, NM_008904 | Ppargc1a | peroxisome proliferative activated receptor, gamma, coactivator 1 alpha | -2.474 | 3.538 |
| 16 | NM_001111288 | Sco2 | SCO cytochrome oxidase deficient homolog 2 (yeast) | -2.163 | 2.252 |
| 17 | NM_013731, NM_001291152,NM_001291154 | Sgk2 | serine/threonine-protein kinase Sgk2 isoform c | -4.149 | 2.101 |
| 18 | NM_001177804,NM_022433,NM_001127351 | Sirt3 | sirtuin 3 | -1.587 | 1.279 |
| 19 | NM_001013780 | Slc25a34 | solute carrier family 25, member 34 | -2.472 | 2.324 |
| 20 | NM_011978 | Slc27a2 | solute carrier family 27 (fatty acid transporter), member 2 | -4.726 | 4.264 |
| 21 | NM_001136260,NM_001197147,NM_018760 | Slc4a4 | solute carrier family 4 (anion exchanger), member 4 | -4.864 | 2.273 |
| 22 | NM_013671 | Sod2 | superoxide dismutase 2, mitochondrial | -1.758 | 1.17 |
| 23 | NM_009463 | Ucp1 | uncoupling protein 1 (mitochondrial, proton carrier) | -3.82 | 1.618 |
| 24 | NM_010247 | Xrcc6 | X-ray repair complementing defective repair in Chinese hamster cells 6 | -2.967 | 2.02 |

**Table S2 Primer sequences for qPCR.**

| Name |  | Sequence(5’→3’) |
| --- | --- | --- |
| *Ucp1* | F | GGATGGTGAACCCGACAACT |
|  | R | AACTCCGGCTGAGAAGATCTTG |
| *Pgc1α* | F | AACAAGCACTTCGGTCATCCCTG |
|  | R | TTACTGAAGTCGCCATCCCTTAG |
| *Prdm16* | F | CAGCACGGTGAAGCCATTC |
|  | R | GCGTGCATCCGCTTGTG |
| *Cidea* | F | GCAGGAACTTATCAGCAAGA |
|  | R | CGTAACCAGGCCAGTTGTGAT |
| *Leptin* | F | ATTTCACACACGCAGTCGGTAT |
|  | R | GGTGAAGCCCAGGAATGAAG |
| *Adipoq* | F | GGCCGTTCTCTTCACCTACG |
|  | R | TGGAGGAGCACAGAGCCAG |
| *Cpt1β* | F | GCACACCAGGCAGTAGCTTT |
|  | R | CAGGAGTTGATTCCAGACAGGTA |
| *Atp5β* | F | GGTTCATCCTGCCAGAGACTA |
|  | R | AATCCCTCATCGAACTGGACG |
| *Cox8b* | F | TGTGGGGATCTCAGCCATAGT |
|  | R | AGTGGGCTAAGACCCATCCTG |
| *Erra* | F | CTCAGCTCTCTACCCAAACGC |
|  | R | CCGCTTGGTGATCTCACACTC |
| *Sirt1* | F | GCAGATTAGTAGGCGGCTTG |
|  | R | TCTCCATCAGTCCCAAATCC |
| *Sirt2* | F | GCCTGGGTTCCCAAAAGGAG |
|  | R | GAGCGGAAGTCAGGGATACC |
| *Sirt3* | F | ATCCCGGACTTCAGATCCCC |
|  | R | CAACATGAAAAAGGGCTTGGG |
| *Nampt* | F | GCAGAAGCCGAGTTCAACATC |
|  | R | TTTTCACGGCATTCAAAGTAGGA |
| *Nads1* | F | ACGGCTGCTCACTACTTGTTA |
|  | R | CTGAGAACCGAGGCAACTTC |
| *Cat* | F | AATCCTACACCATGTCGGACA |
|  | R | CGGTCTTGTAATGG AACTTGC |
| *Gpx3* | F | CCTTTTAAGCAGTATGCAGGCA |
|  | R | CAAGCCAAATGGCCCAAGTT |
| *Sod2* | F | GCACATTAACGCGCAGATCA |
|  | R | AGCCTCCAGCAACTCTCCTT |
| *Idh1* | F | ATGCAAGGAGATGAAATGACACG |
|  | R | GCATCACGATTCTCTATGCCTAA |
| *Idh2* | F | GGAGAAGCCGGTAGTGGAGAT |
|  | R | GGTCTGGTCACGGTTTGGAA |
| *Idh3a* | F | TGGGTGTCCAAGGTCTCTC |
|  | R | CTCCCACTGAATAGGTGCTTTG |
| *Idh3β* | F | TGGAGAGGTCTCGGAACATCT |
|  | R | AGCCTTGAACACTTCCTTGAC |
| *Idh3γ* | F | GGTGCTGCAAAGGCAATGC |
|  | R | TATGCCGCCCACCATACTTAG |
| *Acsm3* | F | CTTTGGCCCCAGCAGTAGATG |
|  | R | GGCTGTCACTGGCATATTTCAT |
| *Cyp2e1* | F | CGTTGCCTTGCTTGTCTGGA |
|  | R | AAGAAAGGAATTGGGAAAGGTCC |
| *Slc27a2* | F | TCCTCCAAGATGTGCGGTACT |
|  | R | TAGGTGAGCGTCTCGTCTCG |
| *Slc4a4* | F | GAAGGTCACCACACGATCTACA |
|  | R | TCCACATCAGATTTGTCGGAGT |
